# The RNA-binding profile of Acinus, a peripheral component of the exon junction complex, reveals its role in splicing regulation

**DOI:** 10.1261/rna.057158.116

**Published:** 2016-09

**Authors:** Julie Rodor, Qun Pan, Benjamin J. Blencowe, Eduardo Eyras, Javier F. Cáceres

**Affiliations:** 1 MRC Human Genetics Unit, Institute of Genetics and Molecular Medicine, University of Edinburgh, Western General Hospital, Edinburgh EH4 2XU, United Kingdom; 2 Donnelly Centre, University of Toronto, Toronto, Ontario M5S 3E1, Canada; 3 Department of Molecular Genetics, University of Toronto, Toronto, Ontario M5S 1A8, Canada; 4 Universitat Pompeu Fabra, E08003, Barcelona, Spain; 5 Catalan Institution for Research and Advanced Studies (ICREA), E08010 Barcelona, Spain

**Keywords:** RNA–protein interactions, RNA-binding proteins, pre-mRNA splicing, exon junction complex, apoptosis

## Abstract

Acinus (apoptotic chromatin condensation inducer in the nucleus) is an RNA-binding protein (RBP) originally identified for its role in apoptosis. It was later found to be an auxiliary component of the exon junction complex (EJC), which is deposited at exon junctions as a consequence of pre-mRNA splicing. To uncover the cellular functions of Acinus and investigate its role in splicing, we mapped its endogenous RNA targets using the cross-linking immunoprecipitation protocol (iCLIP). We observed that Acinus binds to pre-mRNAs, associating specifically to a subset of suboptimal introns, but also to spliced mRNAs. We also confirmed the presence of Acinus as a peripheral factor of the EJC. RNA-seq was used to investigate changes in gene expression and alternative splicing following siRNA-mediated depletion of Acinus in HeLa cells. This analysis revealed that Acinus is preferentially required for the inclusion of specific alternative cassette exons and also controls the faithful splicing of a subset of introns. Moreover, a large number of splicing changes can be related to Acinus binding, suggesting a direct role of Acinus in exon and intron definition. In particular, Acinus regulates the splicing of DFFA/ICAD transcript, a major regulator of DNA fragmentation. Globally, the genome-wide identification of RNA targets of Acinus revealed its role in splicing regulation as well as its involvement in other cellular pathways, including cell cycle progression. Altogether, this study uncovers new cellular functions of an RBP transiently associated with the EJC.

## INTRODUCTION

There is an extensive coupling among different steps in eukaryotic gene expression, as shown by the intimate connection between transcription and RNA processing (Braunschweig et al. 2013; Bentley 2014). In the nucleus, the carboxy-terminal domain (CTD) of the RNA polymerase II large subunit coordinates many RNA processing events by providing a platform for factors involved in different steps of RNA processing (Hsin and Manley 2012). A large variety of RNA-binding proteins (RBPs) bind cellular RNAs to form ribonucleoprotein (RNP) complexes that are central for RNA biogenesis and function (Dreyfuss et al. 2002; Glisovic et al. 2008). RBPs have a profound impact on cellular gene expression networks, affecting processes as diverse as transcription, constitutive and alternative pre-mRNA splicing, microRNA (miRNA) biogenesis and function and mRNA translation. In particular, new protein components of messenger RNA ribonucleoparticles (mRNPs) have been recently identified (Baltz et al. 2012; Castello et al. 2012; Mitchell and Parker 2014).

Some of these RBPs associate with the mRNA in a splicing-dependent manner (Merz et al. 2007). In particular, the exon junction complex (EJC), a multisubunit protein complex, is deposited 20–24 nucleotides (nt) upstream of an exon–exon junction during pre-mRNA splicing and recruits factors involved in NMD, mRNA export, and mRNA localization, acting to link nuclear and cytoplasmic steps in the biogenesis of mRNAs (Le Hir et al. 2000, 2001; Lykke-Andersen et al. 2001). The EJC main core components include four proteins: Magoh, Y14, eIF4A3, and MLN51, which are stably bound to mRNAs and act as landing pads for additional components in both the nucleus and the cytoplasm (Ballut et al. 2005; Andersen et al. 2006; Bono et al. 2006). The EJC remains bound to the mRNA and is only displaced by the translation machinery assisted by the ribosome-associated protein PYM, which acts as an EJC disassembly factor (Gehring et al. 2009; for review, see Le Hir et al. 2015).

Acinus (apoptotic chromatin condensation inducer in the nucleus) was initially identified as a protein required for apoptotic chromatin condensation (Sahara et al. 1999). In addition, several lines of evidence suggested a role for Acinus in RNA processing. First, Acinus was identified as having an RNA recognition motif (RRM), similar to the one present in *Drosophila* Sxl (Sahara et al. 1999) and also a conserved arginine/serine-repeat (RS) domain, a hallmark feature of many defined splicing factors (Boucher et al. 2001). It was subsequently identified as an additional component of the EJC (Tange et al. 2005) and was also shown to be present in a trimeric complex, termed ASAP (for apoptosis and splicing associated protein) that interacts with the EJC (Schwerk et al. 2003; Murachelli et al. 2012). This complex also includes SAP18 (Sin3A-associated protein, 18 kDa), a component of a Sin-3-containing histone deacetylase complex (Zhang et al. 1997), as well as the general activator of pre-mRNA splicing, RNPS1 (Mayeda et al. 1999) and has been shown to have a role in splicing and apoptosis in vitro. In agreement with its presumed role in pre-mRNA splicing, Acinus has also been identified as a component of functional spliceosomes (Rappsilber et al. 2002; Zhou et al. 2002). Additionally, Acinus regulates the splicing of a subset of genes involved in apoptosis in human cells (Michelle et al. 2012) and has been proposed to couple Retinoic acid mediated transcription and splicing (Wang et al. 2015). Interestingly, Acinus, alongside the EJC, is required for definition and excision of neighboring introns in *Drosophila* (Hayashi et al. 2014; Malone et al. 2014). Finally, Acinus was found in the mRNA interactome of proliferating human HeLa cells (Castello et al. 2012). It is thus plausible that Acinus plays a global role in splicing regulation beyond its function as part of the EJC complex. However, genome-wide evidence supporting such a role is lacking.

The CLIP (cross-linking immunoprecipitation) approach with its many variations (including PAR-CLIP, iCLIP, HITS-CLIP) has been successfully used to map in vivo RNA–protein interactions for several RBPs in different experimental systems (for review, see Ascano et al. 2012; Darnell 2012; Kapeli and Yeo 2012; König et al. 2012). Here, we have used the iCLIP variant to identify endogenous RNA targets of Acinus in HeLa cells. This was combined with RNA-seq profiling of Acinus-depleted cells in order to assess the role of Acinus in alternative splicing. We observed that Acinus preferentially binds to protein-coding genes. It is required for the inclusion of specific alternative cassette exons and also controls the splicing of a subset of introns. Importantly, we observed a significant correlation between Acinus binding and the detected splicing changes, suggesting a direct role of Acinus in exon and intron definition. Taken together, our analyses provide evidence for a widespread role for Acinus in pre-mRNA splicing regulation, and in particular for introns harboring suboptimal splice sites.

## RESULTS

### Genome-wide mapping of Acinus binding sites using iCLIP

Consistent with Acinus harboring an RNA recognition motif (RRM), it was recently shown that it crosslinked to RNA as part of the mRNA–protein interactome of HeLa cells (Castello et al. 2012). We confirmed this finding by showing that it binds RNA directly using an mRNA capture assay (data not shown). In order to uncover the roles of Acinus in RNA processing, we decided to focus on the identification of its endogenous RNA targets.

**FIGURE 1.**
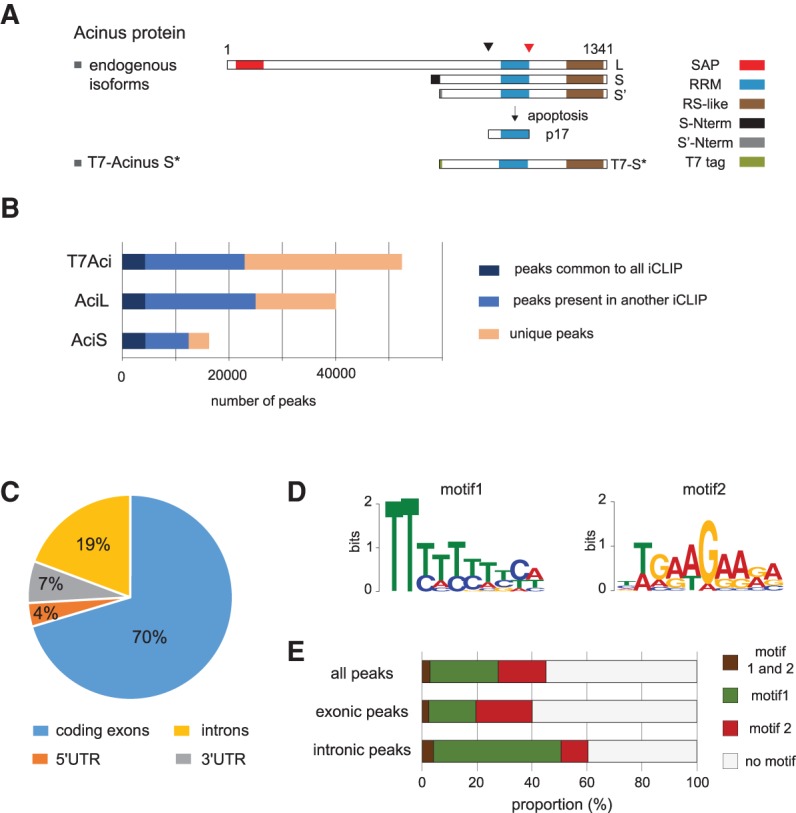
Genome-wide mapping of Acinus RNA-binding sites in HeLa cells using iCLIP-seq. (*A*) Cartoon displaying the three main Acinus protein isoforms (L, S, and S′), the apoptotic-generated p17 fragment, as well as the T7-tagged working construct T7-Acinus S*. Arrows on *top* represent the apoptotic cleavage sites, as previously described (Sahara et al. 1999) (cleavage by Caspase 3 is shown by the red arrowhead, whereas the black arrowhead indicates cleavage by an unknown activity). The different protein domains are highlighted: SAP domain, RNA recognition motif (RRM), RS-like domain, as well as the different N termini of Acinus S and S′ isoforms and the T7 tag. (*B*) Diagram showing the number of peaks for each Acinus iCLIP experiment that are unique to a specific iCLIP experiment or are also present in another experiment. Peaks were detected using Pyicoclip and peaks present in the control experiments were removed from our analysis. (*C*) Distribution of Acinus “common” binding peaks among coding exons, 5′UTR, 3′UTR, and introns. Acinus “common” peaks refer to the 36,271 peaks common to two out of three iCLIP experiments. (*D*) Two significant motifs discovered from the common Acinus peaks using the MEME tool. The search was done for a 6- to 10-nt motif in 20-nt sequences centered on the peak summit from 1000 randomly selected peaks. (*E*) Proportion of peaks from the common data containing motif 1, motif 2, or both motifs. We considered all peaks (*n* = 36,271), exonic peaks (*n* = 25,347), and intronic peaks (*n* = 6944).

The human *ACIN1* gene gives rise to three protein isoforms, termed Acinus-L, Acinus-S′, and Acinus-S, with all containing an RRM domain and a C-terminal RS-like domain (Boucher et al. 2001); however, they differ in their N termini (Fig. 1A). Interestingly, cleavage of Acinus is induced by apoptotic stimuli and mediated by caspase-3 giving rise to a p17 fragment that contains the intact RNA recognition motif (RRM) and promotes chromatin condensation, a hallmark of the terminal stages of apoptosis (Sahara et al. 1999). This cleavage has also been observed in processes involving a nonapoptotic function of caspase 3, such as erythropoiesis (Zermati et al. 2001).

In order to identify the in vivo RNA targets that are common to all Acinus isoforms, we carried out three independent iCLIP experiments in HeLa cells transiently expressing a T7-tagged Acinus construct, which we termed T7-Acinus S*, which contains the RRM domain and the RS-like domain (Fig. 1A) that are present in all three isoforms. Following immunoprecipitation (IP), we observed a specific protein/RNA complex that shifts depending on the RNase concentration (Supplemental Fig. S1A). We isolated this complex and prepared libraries for each iCLIP replicate as well as a matching control library from cells transfected with an empty vector (pCG). We also performed two iCLIP experiments for the endogenous Acinus protein using an antibody that recognizes all three Acinus isoforms (L, S, and S′). We detected two specific signals corresponding to AcinusL/RNA complex and to AcinusS/S′/RNA complexes (Supplemental Fig. S1B). As a control, we performed an immunoprecipitation using rabbit IgG. RNAs were extracted separately for Acinus L and S/S′ complexes and their respective controls. Sequencing data were processed as indicated in the Materials and Methods section and the number of reads for each experiment is shown in Supplemental Figure S1C. We analyzed the correlation between replicates for each different iCLIP experiment, using the read counts obtained per protein-coding gene. For the three iCLIP experiments, we obtained a Pearson correlation above 0.97 between the replicates (Supplemental Fig. S1E,F). Therefore, we decided to pool the replicates for each iCLIP experiment (T7-Acinus S*, Acinus L, and Acinus S iCLIP) before identifying the binding sites. The annotation of the reads revealed that all Acinus isoforms bind mostly protein-coding genes (Supplemental Fig. S2A); however, we noticed that a large number of reads are also derived from repeats and long noncoding RNAs (Supplemental Fig. S2B). For this study, we focused our analysis on the role of Acinus on mRNA biogenesis.

The raw data revealed that Acinus, either the endogenous protein, or the transiently transfected T7-Acinus S*, does not seem to bind discrete positions of a few nucleotides but rather binds to larger regions. Therefore, we decided to identify crosslinked regions using the peak-finding algorithm Pyicoclip from the Pyicoteo tools (Althammer et al. 2011) rather than working at the nucleotide resolution scale (see Materials and Methods). We obtained 52,428 peaks for T7-Acinus S* iCLIP, 40,050 for endogenous Acinus L iCLIP, and 16,240 peaks for Acinus S/S′ iCLIP (Fig. 1B). The difference of peak numbers reflects the difference between mapped read counts. A large number of peaks were detected in at least two of the three iCLIP experiments, suggesting that Acinus L, Acinus S, and the tagged T7-Acinus S* have similar binding affinities (Fig. 1B). We also noticed that some peaks seemed specific to a particular iCLIP experiment. The difference between the tagged-Acinus iCLIP and the endogenous iCLIP can be explained by differences in the efficiency of the iCLIP experiments but also by the overexpression of Acinus protein for the T7-Acinus S* iCLIP. Additionally, it is possible that although Acinus long and short isoforms share target sites, they also have specific binding sites. For instance, Acinus L contains a long N-terminal region with a SAP domain, a putative DNA binding motif (Aravind and Koonin 2000), that could be required for additional roles, and could also influence its RNA binding. However, we noticed that those peaks that are unique for an individual iCLIP experiment are supported by a lower number of reads compared to the peaks common to several iCLIP experiments (Supplemental Fig. S3). For further analysis, we thus defined a list of 36,271 peaks common to two out of three of the iCLIP experiments (see Materials and Methods for details) that will hereafter be referred to as “common peaks.” These common peaks help us define the general binding properties for all Acinus isoforms. We also investigated separately each iCLIP experiment to eventually reveal specific characteristics of each isoform.

### iCLIP annotation and binding motifs

The annotation of the 36,271 common peaks showed that most peak summits fall into exonic coding regions (70%) (Fig. 1C). Peaks were also detected in introns (19%) and to a lesser extent in 5′ and 3′UTRs. The annotation of each individual iCLIP experiment gave a similar distribution pattern (Supplemental Fig. S4).

Using MEME (Bailey et al. 2009), we searched for motifs enriched in the iCLIP data, taking 20-nt sequences centered on the peak summit. Using sequences from 1000 randomly selected common peaks (see Materials and Methods), we obtained two significant motifs (Fig. 1D). Around 45% of Acinus peaks contain one of these two motifs (Fig. 1E). Motif 1 is a T-rich sequence, whereas motif 2 is a “GAAGAA”-like motif. Interestingly, motif 1, which resembles the polypyrimidine tract found in the vicinity of 3′ splice sites, was found to be enriched in peaks located in introns (Fig. 1E). In contrast, motif 2, which is similar to the consensus for a strong exonic splicing enhancer sequence, and the binding site of several serine/arginine-rich (SR) proteins (Tacke et al. 1998; Sanford et al. 2008, 2009; Änkö et al. 2012; Pandit et al. 2013; for review, see Long and Caceres 2009; Howard and Sanford 2015), was preferentially found in exons (Fig. 1E). Importantly, a similar motif was also found to be associated with the binding site of the EJC (Saulière et al. 2012; Singh et al. 2012). A search for motifs was also done for each iCLIP individually and gave similarly enriched motifs (Supplemental Fig. S4B). Altogether, these data seem to exclude specificity of binding based on a particular isoform of Acinus.

### Acinus binds the canonical EJC position in association with eIF4A3

**FIGURE 2.**
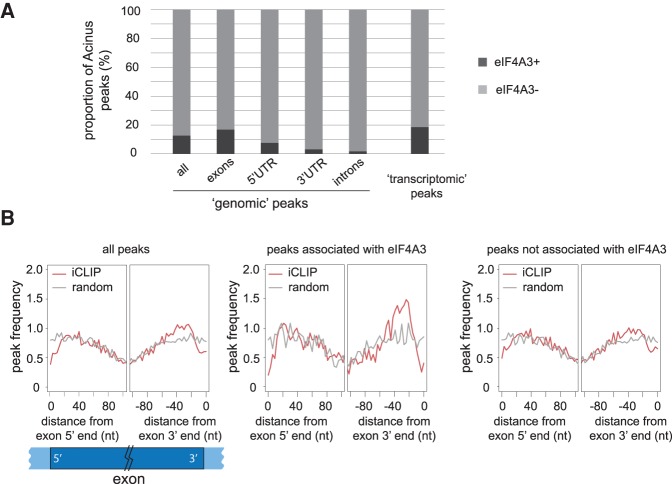
Proportion of Acinus peaks associated with eIF4A3 peaks and enrichment at “canonical” EJC occupancy site (24-nt upstream of an exon junction). (*A*) Proportion of Acinus peaks found in close proximity of eIF4A3 peaks (eIF4A3+) (maximum distance of 10 nt between both peak summits). The analysis was done on “genomic” peaks (reads mapped to the genome) depending on their location: all peaks (*n* = 36,271), exonic peaks (*n* = 25,347), 5′UTR peaks (*n* = 1347), 3′UTR peaks (*n* = 2379), and intronic peaks (*n* = 6944), but also on “transcriptomic” peaks (reads mapped to a representative transcriptome data set) (*n* = 13,231). eIF4A3 CLIP-seq data described in Saulière et al. (2012) were downloaded from GEO: GSM1001331. (*B*) Distribution of the peak frequency according to the distance of the peak summit from the exon 5′ end or the exon 3′ end. This analysis was done using Acinus’ “common transcriptomic” peak data for all peaks (*n* = 10,735), peaks associated with eIF4A3 (*n* = 2000), or peaks not associated with eIF4A3 (*n* = 8735) (from *left* to *right*). Only internal exons were used. The iCLIP data are represented by a red line. The gray line corresponds to randomized peak summit positions.

Acinus was described as an auxiliary component of the EJC (Tange et al. 2005) and preferentially binds to exons, as is the case with eIF4A3 (Sauliere et al. 2012). To investigate if Acinus always binds RNA in association with the EJC, we used the available eIF4A3 CLIP data (Saulière et al. 2012), which was also carried out in HeLa cells. We analyzed these data in a similar way as our Acinus iCLIP (see Materials and Methods) and obtained 88,152 peaks. We then looked for eIF4A3 peaks in close proximity to Acinus peaks. Since Acinus and eIF4A3 data were obtained using different variations of the CLIP protocol, we tolerated a 10-nt distance between the two peak summits to consider an association between the binding of both proteins. This analysis revealed that only 13% of Acinus peaks are in close proximity to an eIF4A3 binding site (Fig. 2A). This difference could be due to the fact that Acinus and eIF4A3 data were obtained using different variants of the CLIP protocol. An alternative possibility is that Acinus binds RNAs independently of the EJC. Interestingly, the proportion of Acinus peaks associated with eIF4A3 is higher in exons (17%), where the EJC components are deposited, and very low (2%) in introns (Fig. 2A). Accordingly, a search for motifs in Acinus peaks associated with eIF4A3 recovered the “GAAGAA” motif 2 but not motif 1 (data not shown). This comparative analysis of Acinus and eIF4A3 CLIP experiments was done on reads mapped to the genome. While the mapping to the genome is suitable for proteins binding to pre-mRNAs, this approach is inadequate if the protein binds to spliced RNAs, in proximity to the exon–exon junction. Indeed, reads spanning the exon–exon junction do not map to the genome. To avoid this bias, we mapped Acinus and eIF4A3 iCLIP reads to the spliced transcriptome, rather than to the genome, and identified peaks with Pyicoclip. Again, we looked for the presence of eIF4A3 peaks in close proximity of Acinus peaks and observed that around 19% of “common transcriptomic” peaks were defined as eIF4A3+ (Fig. 2A). As eIF4A3 binds mRNA at “canonical” exon junction complex site (24-nt upstream of the exon–exon junction), as well as to “noncanonical” sites (Saulière et al. 2012; Singh et al. 2012), we analyzed Acinus preferential binding sites within exons using the peaks obtained from the transcriptomic mapping. A small increase of peak density was observed 20- to 40-nt upstream of exon–exon junctions (Fig. 2B, left panel). Moreover, focusing on Acinus peaks associated with eIF4A3 peaks, even though the signal is not as sharp as before due to the lower number of peaks, we observed a strong enrichment between −20 and −40 nt (Fig. 2B, middle panel). Importantly, these data suggest that Acinus is preferentially bound at the EJC “canonical” site, when associated with eIF4A3. In agreement, Acinus peaks not overlapping eIF4A3 bound sites show a strong decrease of the −24-nt enrichment (Fig. 2B, left panel). Analysis of individual iCLIP data shows similar profiles (Supplemental Fig. S5A–C). The enrichment upstream of the exon junction is obvious for peaks associated with eIF4A3 and is decreased (or even lost completely for Acinus S and T7 Acinus S* iCLIP) when focusing on eIF4A3-negative peaks (Supplemental Fig. S5A–C). Interestingly, a similar analysis for eIF4A3 peaks revealed that the observed eIF4A3 enrichment upstream of the exon–exon junction does not depend on its association with Acinus peaks (Supplemental Fig. S6). These observations fit a model whereby all EJCs at canonical sites are composed of the core components (eIF4A3, MAGOH, Y14, and MLN51) but have a different composition of the peripheral factors, such as Acinus.

### Acinus binding to intronic regions

**FIGURE 3.**
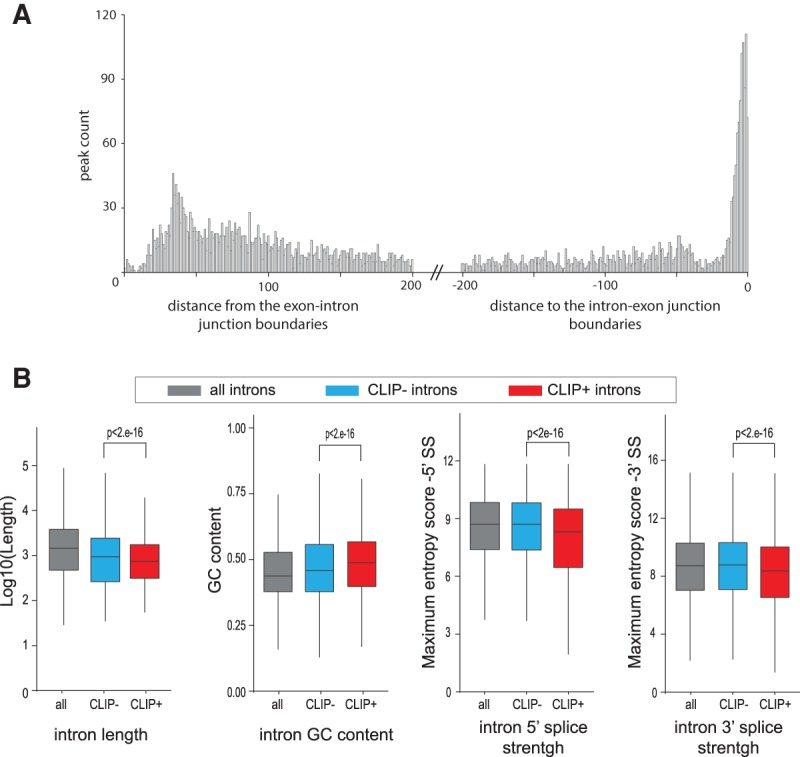
Acinus binds introns with weak splice sites close to the intron boundaries. (*A*) Histogram showing the number of Acinus common peaks in introns depending on the distance of the peak summit from and to the exon–intron junction boundary. (*B*) Box plot of the intron length (Log_10_), GC content, 5′ splice strength, and 3′ splice strength for all (*n* = 129,270), CLIP− (*n* = 23,654), and CLIP+ (*n* = 4838) introns. Acinus CLIP+ and CLIP− introns were defined according to a read count approach. The splice strength was calculated using the maximum entropy model (Yeo and Burge 2004). The *P*-value evaluated with the two-sided Wilcoxon rank-sum test is indicated.

Mapping of iCLIP peaks showed that Acinus also binds introns (Fig. 1C). Interestingly, Acinus copurifies with spliceosomal complexes A and C (Rappsilber et al. 2002; Zhou et al. 2002); thus, it could be recruited to introns during pre-mRNA splicing dependently or independently of the EJC. Using the peaks detected in the common iCLIP data, we identified 3792 introns bound by Acinus. The analysis of the position of the peak summits within introns showed an enrichment of Acinus peaks in a 25- to 100-nt region downstream from the exon–intron boundaries (Fig. 3A). Acinus peak summits are also highly enriched in the 3′ side region of the intron, which contains the polypyrimidine tract and the 3′ splice site. This enrichment is observed at the border of the intron, suggesting that Acinus peaks span the intron–exon boundary. A similar profile was observed when independently analyzing individual iCLIP experiments (Supplemental Fig. S7). Importantly, the identified “T-rich” binding motif (motif 1, Fig. 1D) is consistent with Acinus binding to the polypyrimidine tract region. Interestingly, T-rich (or U-rich in pre-mRNA) motifs are also often found downstream from the 5′ splice site (Aznarez et al. 2008) where Acinus binds. In contrast, eIF4A3 peaks in introns do not show any enrichment close to the exon–intron boundaries (Supplemental Fig. S7D), suggesting that Acinus binding in introns does not occur in association with the core exon junction complex.

As Acinus binds both pre-mRNAs and mRNAs, we thought that the relative abundance between pre-mRNAs and mRNAs could underestimate our intron binding estimation. We also noticed that some introns contain a consistent number of reads but as they do not define a clear peak, they are lost in the analysis. Therefore, we decided to use a read-count analysis to define a complete list of introns bound by Acinus (Supplemental Table S1). We defined CLIP+ introns as those with a fivefold enrichment of read counts between Acinus iCLIP and control, or with a minimum of five reads in the Acinus iCLIP data. While the peak data analysis gave us 3792 introns with Acinus binding site, this read-count approach identified 22,813 introns defined as CLIP+ in two out of three iCLIP experiments. Moreover, we found 4838 common CLIP+ introns for the three iCLIP data sets (AciL, AciS, and T7Aci) (out of 129,270 introns) (Supplemental Table S1). To investigate whether Acinus-bound introns exhibit specific functional properties, we also defined a list of 23,654 CLIP- (CLIP negative) introns that contain a number of reads in all Acinus iCLIPs less or equal to the number of reads in the control iCLIPs. The comparison between CLIP− and CLIP+ introns revealed that introns bound by Acinus are significantly shorter, have a higher GC content, and display weaker 5′ and 3′ splice sites (Fig. 3B). This analysis suggests that Acinus bound to introns could be involved in splicing regulation independently of the EJC.

### A role for Acinus in alternative splicing

**FIGURE 4.**
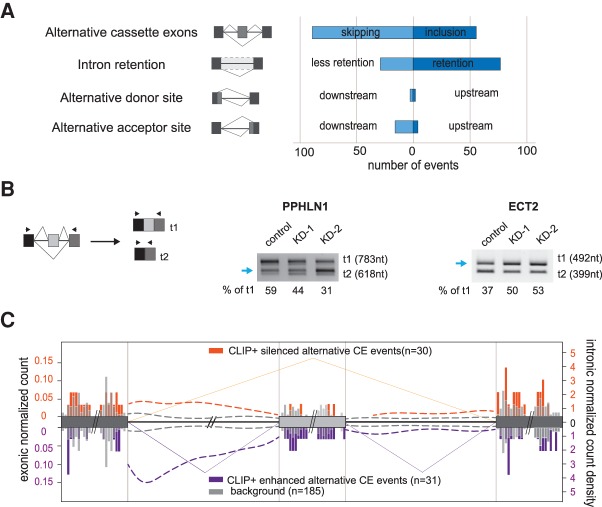
Splicing regulation mediated by Acinus. (*A*) Summary of splicing changes following Acinus depletion, as revealed by RNA-seq analysis. The graph shows the number of events in the different splicing categories, as well as the direction of those changes. (*B*) Validation of alternative cassette exon for the PPHLN1 and ECT2 transcripts in Acinus-depleted cells using two distinct siRNAs. The blue arrow indicates the isoform showing an increase in Acinus-depleted cells. The proportion of transcript t1, corresponding to the longest isoform, was estimated after quantification of the transcript's abundance by an Agilent Bioanalyzer. (*C*) An RNA map of Acinus binding sites within regulated alternative cassette exons. Data for Acinus silenced exons (*n* = 30) are shown in orange, data for Acinus enhanced exons (*n* = 31) are in purple, and the control events (*n* = 185) are in gray. Acinus iCLIP common peaks were used for this analysis. The exonic normalized counts are shown as a histogram (bin interval 5 nt) for regions 50-nt downstream or upstream the splice site. The distribution of the relative distance in introns is shown as a normalized count density (dotted lines).

In order to analyze globally the effects of Acinus on constitutive and alternative splicing, we performed RNA-seq on poly(A)^+^ RNA isolated from HeLa cells following siRNA knockdown of all three Acinus isoforms. Knockdown was performed in triplicate using an Acinus siRNA together with three independent control experiments using a nontargeting siRNA (Supplemental Fig. S8A). Independent knockdowns and controls clustered together in our gene expression analysis (Supplemental Fig. S8B). In Acinus-depleted cells, we found 408 up-regulated and 382 down-regulated genes with a twofold change and an adjusted *P*-value of 0.05 (Supplemental Table S2). We also quantified all alternative splicing events in each RNA-seq data in an unbiased way considering all hypothetically possible splice junctions from annotated and de novo splice sites, as previously described (Irimia et al. 2014). Using a very stringent filtering criteria (see Materials and Methods), we detected changes for all types of alternative splicing events: 145 alternative cassette exons, 106 intron retention (IR) events, as well as 5 and 20 events for alternative donor and acceptor sites, respectively (Fig. 4A; Supplemental Table S3). Interestingly, those changes show enrichment toward one specific direction of the splicing change (Fig. 4A). For instance, upon Acinus depletion, we observed a significant switch toward cassette exon skipping, as compared to exon inclusion (χ^2^ test 0.006). This would indicate that Acinus is required for the inclusion of those alternative exons. As for IR events, the bias toward an increase in retention of introns in Acinus-depleted cells was striking (χ^2^ test 3.10–6). We validated several of these splicing changes using the same siRNA used for the RNA-seq data (knockdown [KD-1]) and also with another siRNA (KD-2), which gave a comparable depletion of Acinus protein (Supplemental Fig. S8C). We validated the changes observed for the alternative cassette exons in PPHLN1 and ECT2 transcripts that showed a reciprocal response to Acinus depletion (Fig. 4B). Periphilin 1 (PPHLN1) protein is part of a transcriptional co-repressor complex and has been involved in epithelial differentiation whereas ECT2 is a guanine nucleotide exchange factor and transforming protein (Miki et al. 1993; Kazerounian and Aho 2003).

In order to investigate whether the effect of Acinus on alternative exons was direct, we used the iCLIP data to draw an RNA map (Fig. 4C; Witten and Ule 2011). From the 145 affected cassette exons, we detected Acinus binding peaks in proximity of the events for 30 included cassette exons and 31 skipped cassette exons. As a control, we selected 185 alternative cassette exons with Acinus peaks but that were not affected by Acinus depletion. The peak distribution in exons revealed that Acinus binds to the surrounding constitutive exons for both enhanced and silenced cassette exon events with some small difference compared to the control set. We also observed increased binding to the cassette exon itself for Acinus enhanced alternative exons. Interestingly, the peak distribution in introns shows that Acinus strongly binds the upstream introns of enhanced alternative exon events, with an enrichment downstream from the 5′ splice site. Acinus binding in introns is also higher compared to the control in the upstream introns of silenced exon events as well as in the downstream introns of both silenced and enhanced exons.

### Acinus directly regulates splicing of a subset of introns

**FIGURE 5.**
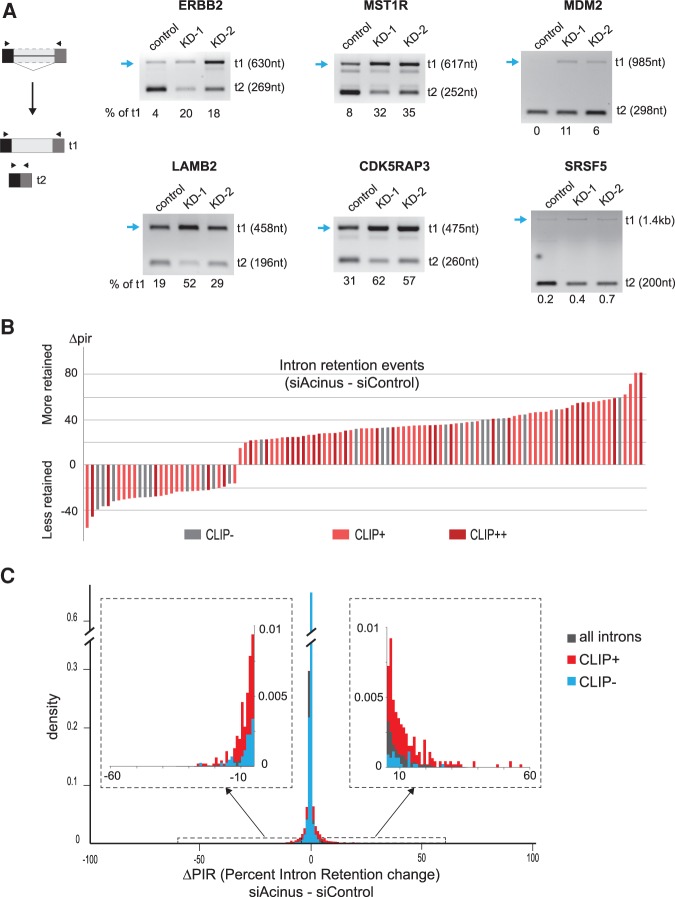
Intron retention changes mediated by Acinus. (*A*) Validation of intron retention changes for the ERBB2, MST1R, MDM2, LAMB2, CDK5RAP3, and SRSF5 transcripts in Acinus-depleted cells using two distinct siRNAs. The blue arrow indicates the isoform showing an increase in Acinus-depleted cells. The proportion of transcript t1, corresponding to the longest isoform, was estimated after quantification of the transcript's abundance by an Agilent Bioanalyzer. (*B*) Plot showing the Δpir (difference of intron retention level) for the intron retention events. Introns bound by Acinus are in red (CLIP+: in two out of three Acinus iCLIP and CLIP++: in all three iCLIP experiments). (*C*) Histogram showing the density of introns depending on their change in intron retention level (Δpir) in the RNA-seq data. The analysis was done on introns with reliable intron retention levels: 89,752 for all introns, for 4557 CLIP+ introns, and for 4461 CLIP− introns. The *inside* panels are zoomed-in views of the plot for a Δpir in the ranges (−60, −5) and (5:60).

Next, we validated the increase in intron retention for the pre-mRNAs of three proto-oncogenes, ERBB2, MST1R (also known as RON), and MDM2, as well as for those encoding laminin β 2 (LAMB2), the CDK5 regulatory subunit-associated protein 3 (CDK5RAP3), and the splicing factor SRSF5 (Fig. 5A). In every case, we noticed an increase in intron retention upon decreased levels of Acinus, suggesting that Acinus controls the splicing of those introns. In the case of SRSF5, there is a clear reduction in the levels of the isoform lacking the intron, which is not reflected in an obvious increase in the intron-containing isoforms, strongly suggesting that this isoform is being down-regulated by NMD, as was previously suggested (Änkö et al. 2012). As our analysis of the RNA-seq data revealed a prominent effect of Acinus in intron retention, we decided to assess whether this effect is related to a direct binding of Acinus to those regulated introns. Using the iCLIP data, we found that introns significantly affected by Acinus are enriched for CLIP+ introns (one-sided Fisher test, *P*-value < 0.01). Indeed, 27 out of 106 introns with IR changes were defined as CLIP+ in the three Acinus iCLIP experiments, while 82 introns are CLIP+ in at least two iCLIP data sets (Fig. 5B).

Next, we investigated the relationship between direct binding of Acinus to an intron and the corresponding IR changes upon Acinus depletion. We compared the distribution of changes in IR level after Acinus depletion for all, CLIP+ or CLIP− introns (Fig. 5C). For this analysis, we only kept introns whose intron retention level could be reliably estimated in all RNA-seq samples and calculated the Δpir that corresponds to the change in IR level between Acinus depletion and control. The retention level of most introns is not affected by Acinus depletion as revealed by the high density of introns around the zero axis. However, we observed that the distribution of CLIP+ introns is significantly shifted away from this zero axis compared to CLIP− introns (*P* = 3.4 × 10^−7^, Mann-Whitney *U*-test). This shows that CLIP+ introns have a higher proportion of introns presenting a change in their IR level (Δpir above and below five) and preferentially an IR retention level (Δpir above five). The link between Acinus binding in intron and intron splicing defect is perfectly illustrated for genes with more than one affected intron. For example, we observed a correlation between the number of reads in each intron and the level of intron retention change for the LAMB2 and ZMIZ2 transcripts (Supplemental Fig. S9). Altogether, these data indicate a direct role of Acinus in controlling the splicing of a subset of introns.

### Acinus and eIF4A3 splicing co-regulation

It was reported that EJC core proteins have a role in the regulation of alternative splicing in HeLa cells (Wang et al. 2014). In the same study, it was noticed that the validated EJC-dependent splicing events were not affected by Acinus depletion. However, the co-regulation between Acinus and the EJC was not investigated globally and was only focused on alternative cassette exons. In contrast, in *Drosophila*, the EJC as well as Acinus co-regulate the intron removal of a subset of introns (Hayashi et al. 2014). We decided to investigate whether those splicing events that were regulated by Acinus were also co-regulated by the core EJC component, eIF4A3. We used the available RNA-seq data obtained in HeLa cells following depletion of eIF4A3 in duplicate (Wang et al. 2014). To compare easily with Acinus splicing changes, we reanalyzed the data using the same pipelines used for Acinus RNA-seq. As the experiments were carried out under different conditions, the direct comparison of the differential changes after stringent filtering was not really informative. Instead, we studied all the significant changes upon Acinus or eIF4A3 depletion and used a Δpsi of 10 to consider a change for the splicing events (see Materials and Methods). We found a large proportion of co-regulated events for alternative exon skipping and increased intron retention (Supplemental Fig. S10A). Interestingly, for the increased intron retention events, most of the events depend on both Acinus and eIF4A3 proteins and only a minimal number of events seem to be regulated by Acinus (or eIF4A3) only. We validated a few events experimentally by RT-PCR upon siRNA-mediated depletion of eIF4A3 (Supplemental Fig. S10C). These data suggest that Acinus and the EJC co-regulate a subset of alternative splicing events.

### Role of Acinus in pre-mRNA splicing and apoptosis

**FIGURE 6.**
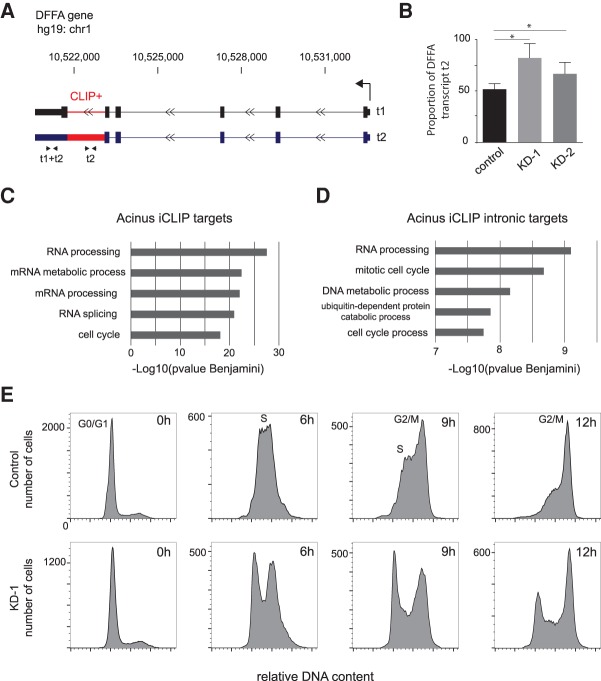
Acinus regulates intron retention in the DFFA pre-mRNA, which encodes an apoptotic regulator, as well as of pre-mRNAs encoding RNA processing factors and cell cycle regulators. (*A*) Structure of DFFA transcripts. The transcription start site and the direction of transcription are indicated by an arrow. The CLIP+ intron (retained in transcript 2) is indicated in red. Arrows *underneath* indicate primers used for RT-qPCR analysis to detect specifically transcript 2 or both transcripts t1 and t2. (*B*) Ratio of DFFA transcript 2 compared to total DFFA transcript. The quantification of transcript 2 and total transcript was done by RT-qPCR using specific primers. The quantification was done on four replicates for control cells and five replicates for knockdown 1 and 2 of Acinus. (*) *P*-value below 0.05 as calculated using the Mann-Whitney test. (*C*) Graph showing selected enriched GO terms for genes bound by Acinus (4416 genes). This analysis was done using DAVID functional annotation tool and ranked using the Benjamini corrected *P*-value. (*D*) Graph showing selected enriched GO term for genes that show binding of Acinus in introns (2668 genes). This analysis was done using DAVID functional annotation tool, as described above. (*E*) Cell cycle profile of control and Acinus-depleted HeLa cells following release from a G1 arrest at 0, 6, 9, and 12 h. After siRNA treatment, cells were arrested in G1 using 2 mM mimosime treatment for 20 h. After release, cells were collected at different time points. The cell cycle profile was obtained by propidium iodide staining and flow cytometry.

Acinus was initially described to be required for apoptotic chromatin condensation (Sahara et al. 1999; Hu et al. 2005). We searched our iCLIP and RNA-seq data sets for RNAs bound by Acinus that were reported to be involved in the apoptotic pathway. Interestingly, we observed that Acinus binds the DFFA transcript, also known as ICAD, a major regulator of DNA fragmentation during apoptosis (Nagata 2000). The DFFA gene produces two main transcripts generated by alternative splicing that only differ by the retention of the last intron (Fig. 6A). Since Acinus binding was specifically detected in the last intron of the DFFA gene (as shown by the read count iCLIP approach), we hypothesized that this binding could be important to regulate the splicing of this intron and this IR event did show a 15% increase after Acinus depletion in our RNA-seq data analysis. The level of the DFFA-2 transcript, containing the retained introns, as well as the level of total transcripts, was further evaluated by RT-qPCR in control and Acinus-depleted HeLa cells. This analysis verified an increase in the proportion of the transcript containing the retained intron after Acinus depletion (Fig. 6B).

Importantly, the observed increase in intron retention upon Acinus depletion, together with binding to this intron, suggested a direct role for Acinus in this regulated AS event. The DFFA transcript containing the retained intron encodes a shorter DFFA protein isoform (DFFA-S/ICAD-S), which does not work as a chaperone for the caspase-activated DNase (DFFB or CAD) and consequently impairs the DNA fragmentation induced by apoptotic stimuli (Li et al. 2005). The splicing changes toward the DFFA-S protein isoform observed in Acinus-depleted cells can thus help to explain the previously observed DNA fragmentation inhibition due to Acinus loss (Joselin et al. 2006; Rigou et al. 2009).

### Cellular pathways regulated by Acinus

In order to search for other cellular functions of Acinus, we performed a Gene Ontology (GO) term enrichment analysis of the 4416 protein-coding genes bound by Acinus (from the list of 36,271 common peaks). We also analyzed separately genes that show Acinus binding in introns (2668 genes). We noticed enrichment for RNA processing, mRNA metabolic processes as well as pre-mRNA splicing pathways (Fig. 6C,D). For some of these targets, Acinus controls their alternative splicing as revealed by changes detected by RNA-seq (Supplemental Table S3). For example, Acinus regulates an alternative cassette exon in the pre-mRNA encoding the mRNA export factor THOC2, as well as an AS cassette exon and an IR event in the pre-mRNA encoding the transcription termination factor TTF2 (Supplemental Table S3). We validated the impact of Acinus depletion on the retention of a specific intron for the splicing factor SRSF5 (Fig. 5A). The involvement of Acinus in the regulation of other RNA processing genes confirms the idea that RPBs are part of a cross-regulatory posttranscriptional network (Sanford et al. 2009; Kosti et al. 2012; Shankarling et al. 2014; for review, see Kalsotra and Cooper 2011).

Surprisingly, Acinus also binds many RNAs encoding proteins involved in cell cycle regulation (Fig. 6C,D). Among them, we validated IR changes observed for CDK5RAP3 and MDM2 transcripts, both involved in cell cycle progression (Bartek and Lukas 2001; Jiang et al. 2009) (as shown on Fig. 5A). While eIF4A3 targets were also enriched for RNA processing genes, an enrichment for cell cycle genes was not observed (Saulière et al. 2012). Cell cycle defects have been previously observed in K562 cells following Acinus depletion (Jang et al. 2008). Following mimosine-induced G1 cell cycle arrest and release, control cells progressed through the cell cycle reaching G2 phase after 12 h. In contrast, a large proportion of cells that have been depleted of Acinus did not enter S phase and remain blocked in G1. Similar results were obtained by knocking down Acinus with a different siRNA (data not shown), suggesting a role for Acinus in cell cycle progression.

## DISCUSSION

Here, we used the iCLIP protocol to identify endogenous RNA targets for Acinus, an auxiliary component of the EJC, in human cells. We combined this with full transcriptome analysis of gene expression and splicing changes upon Acinus depletion and demonstrated a clear role for Acinus in splicing regulation. The iCLIP experiments identified RNA targets associated with all isoforms of Acinus, including the long isoform (Acinus-L) and the two short isoforms (Acinus S/S′). In addition, we also performed another iCLIP experiment with an overexpressed T7-tagged version of Acinus (T7-S*), which comprises a region that is common to all three isoforms, including the RRM and the RS-like domains (Fig. 1A). Our analysis of “common” peaks (present in two out of three iCLIP experiments) represents a stringent picture of the Acinus binding profile. The iCLIP for individual Acinus isoforms revealed similar RNA-binding properties for Acinus L and Acinus S/S′ as well for the common T7-Acinus S* isoforms. Acinus binds strongly to exons, in agreement with its identification as a component of the EJC (Tange et al. 2005). We compared Acinus iCLIP with the CLIP of eIF4A3 (Saulière et al. 2012). Surprisingly, only a small proportion of Acinus peaks are close to eIF4A3 peaks, suggesting that Acinus and eIF4A3 do not always bind to RNA in close proximity (Fig. 2). Nonetheless, our data confirm that Acinus is a peripheral EJC component, since it is only detected at “canonical” EJC sites almost exclusively in association with eIF4A3. Conversely, the presence of eIF4A3 at “canonical” EJC sites does not depend on the presence of Acinus.

We show that Acinus binds mostly protein-coding transcripts; however, we also detected reads coming from noncoding RNAs, preferentially repeats and long noncoding RNAs, suggesting a putative role for Acinus in their regulation. Importantly, we also detected Acinus peaks in introns downstream from the 5′ splice site as well as spanning the 3′ splice site. We obtained a more complete list of introns bound by Acinus by using a read count approach. This analysis revealed that Acinus preferentially binds to suboptimal introns (Fig. 3).

### A role for Acinus in splicing regulation

RNA-seq analysis revealed that Acinus depletion leads to several alternative splice changes in HeLa cells. Importantly, the combination of iCLIP and RNA-seq data suggested a direct role for Acinus in splicing regulation (Fig. 4). Acinus seems to regulate the inclusion of alternative cassette exon by binding to the exon itself but also by binding the upstream intron. We also observed a significant number of intron retention changes following Acinus depletion in HeLa cells. Similarly, intron retention defects were previously observed in *Drosophila* following Acinus knockdown (Hayashi et al. 2014) showing that this regulation is conserved among species. Interestingly, we show a clear correlation between the observed IR changes and a direct binding of Acinus, highlighting the requirement of Acinus for the faithful splicing of a subset of introns.

Here, we focused on the role of Acinus in splicing regulation; however, it remains possible that Acinus could be involved in other nuclear RNA processing steps in connection or independently of its association with the EJC. A proposed role for Acinus in mRNA export of cellular RNAs has been proposed (Chi et al. 2014); however, the direct contribution of Acinus in this process and the transcript specificity of this effect has not been determined. Intron retention (IR) has not been considered as a prevalent alternative splicing event in higher eukaryotes; however, it was recently demonstrated that it is frequent in mammals acting as a mechanism to reduce the levels of transcripts in a cell-type or tissue-specific manner, fine-tuning the transcriptome (Braunschweig et al. 2014). Transcripts harboring retained introns are generally retained in the nucleus and this could indirectly account for the observed role of Acinus in mRNA export (Chi et al. 2014). They could be also targeted for degradation in the nucleus, and those that are exported are usually degraded in the cytoplasm by the NMD machinery as they often contain a premature stop codon (Ge and Porse 2014). Intron retention regulation has been observed in different cell types (Braunschweig et al. 2014), during specific differentiation processes such as erythropoiesis (Pimentel et al. 2016) or granulocyte differentiation (Wong et al. 2013). Intron retention also plays a role in cancer as a mechanism of tumor suppressor inactivation (Jung et al. 2015). Since Acinus controls the splicing of a subset of introns, it could represent a good candidate for the specific control of IR in a precise biological context, as was observed for hnRNPLL in intron retention regulation in T cells (Cho et al. 2014). However, contrary to the known tissue-specific expression of hnRNPLL, the *ACIN1* gene appears to be ubiquitously expressed (data not shown). There could be, nonetheless, physiological conditions upon which levels of Acinus could be altered. For instance, hypermethylation of this gene has been observed in early stage lung adenocarcinoma, which could lead to a decrease in its expression (Shu et al. 2006), and Acinus cleavage by caspase-3 has been observed upon apoptotic stimuli (Sahara et al. 1999) but also during erythropoiesis (Zermati et al. 2001). Importantly, there are other nonapoptotic functions of caspase 3, such as cell proliferation and differentiation, which could potentially lead to Acinus cleavage (Schwerk and Schulze-Osthoff 2003). Interestingly, intron retention was detected during terminal erythropoiesis (Pimentel et al. 2016), a pathway where Acinus cleavage was observed (Zermati et al. 2001). This raises the attractive hypothesis that Acinus could be involved in the regulation of some of the regulated intron retention events. Future work will be required to investigate the role of Acinus down-regulation under different physiological conditions leading to alterations in alternative splicing, including retention of bound introns.

### eIF4A3-dependent and independent splicing events regulated by Acinus

The exon junction complex (EJC) is a dynamic complex and while most exon–exon junctions are apparently bound by the four core proteins, the composition of peripheral factors is supposed to be variable, depending on the different stage of the mRNA life and maybe depending on the exon junction position within the same transcript (Le Hir et al. 2015). The dynamics of EJC composition has not been studied so far in a transcriptome-wide manner. Some studies have addressed the role of the EJC in constitutive and alternative splicing. In *Drosophila*, the EJC was reported to be required for the splicing of long intron-containing genes, particularly affecting the RAS/MAPK signaling pathway (Ashton-Beaucage et al. 2010; Roignant and Treisman 2010). In mammalian cells, knockdown of EJC core proteins results in widespread alternative splicing changes, with similar changes in splicing observed upon knockdown of different EJC core components, yet displaying differences with down-regulation of other splicing factors that are not components of the EJC (Wang et al. 2014). A role for EJC components in the regulation of splicing of apoptotic regulators has also been reported (Michelle et al. 2012). EJCs have been shown to multimerize with one another and form higher-order complexes containing in particular numerous SR proteins, leading to a model whereby EJCs and SR proteins could work in cooperation to promote mRNA packaging and compaction, affecting pre-mRNA splicing (Singh et al. 2012). Surprisingly, we observed that the core EJC factor, eIF4A3, which shows a different RNA-binding profile compared to Acinus, particularly in introns, co-regulates some of Acinus regulated splicing events, mainly related to IR events (Supplemental Fig. S10). This is in agreement with previous findings in *Drosophila* where the core EJC plus the accessory factors RnpS1 and Acinus promote efficient splicing of neighboring introns (Hayashi et al. 2014). Here, EJC-dependent splicing was proposed as a mechanism to control the temporal order of splicing events. However, in all these studies, the direct link between the EJC binding pattern and the observed splicing defect was not investigated or was not conclusive. The fact that Acinus, but not eIF4A3, binds to introns close to the exon–intron boundaries suggests a unique role of Acinus in splicing regulation that is different from the EJC. This leads to a possible model whereby the deposition of the EJC will subsequently recruit Acinus to the partially spliced pre-mRNA to control the splicing of suboptimal introns. The observed low association between Acinus and eIF4A3 peaks only reveals that both proteins do not have the same binding sites on the RNA, but this does not exclude the possibility that the EJC with eIF4A3, at either canonical or noncanonical sites, interacts with and/or recruits Acinus to the exon–intron junction.

The genome-wide RNA-binding profile of Acinus is also suggestive of additional cellular functions. Supporting this idea, the GO-term enrichment for Acinus (this study) and eIF4A3 mRNA targets (Saulière et al. 2012) revealed significant differences. Whereas both proteins regulate genes involved in RNA processing, only Acinus RNA targets are enriched in genes with a role in cell cycle regulation. Our analysis in HeLa cells as well as previous data in leukemia cells (Jang et al. 2008) revealed a cell cycle G1 arrest phenotype in Acinus-depleted cells. In contrast, the core proteins of the EJC Y14 (Ishigaki et al. 2013) and Magoh (Silver et al. 2013) are required for M phase progression. Therefore, Acinus could be involved in the control of mRNA processing during specific cell cycle stages.

We discovered that Acinus binds and regulates the splicing of the last intron of the DFFA/ICAD pre-mRNA, giving rise to the production of DFFA-L (transcript 1, also known as ICAD-L) that has a function as chaperone for the caspase-activated DNase (DFFB/CAD) and promotes DNA fragmentation upon apoptotic stimuli (Liu et al. 1997; Enari et al. 1998). As this splicing event was also shown to be regulated by the SR protein SRSF1 (Li et al. 2005), it would be worth investigating further whether Acinus and SRSF1 cooperate in this regulation.

In summary, we present here a genome-wide RNA-binding landscape for the peripheral EJC factor Acinus that reveals a crucial role of Acinus in the regulation of splicing events co-regulated by the EJC. The identified mRNA targets place Acinus within a cross-regulatory RNA processing network and support its role in cell cycle progression.

## MATERIALS AND METHODS

### Cell culture

HeLa cells were cultured in medium (DMEM, 10% FCS, 1% penicillin/streptomycin) at 37°C in an atmosphere containing 5% CO_2_.

### Constructs, transfections, and siRNA-mediated depletions

The pCG-T7 Acinus S* construct was created by cloning the sequence that is common to the three main Acinus isoforms (nt 162–1889 from *Homo sapiens* acinusS mRNA, complete cds, GenBank: AF124727.1) into the mammalian expression vector, pCGT7, which was previously described (Cáceres et al. 1997). Of note, 70%–90% confluent cells were transfected with the pCG-T7 Acinus S* vector using Lipofectamine 2000 (Invitrogen). Expression of the tagged protein was detected by Western blot analysis 24 h after transfection. Depletion of Acinus was achieved after two rounds of transfection using Dharmafect Reagent 1 (Dharmacon). siRNA1 (CUGCAGAGCAUGAAGUAAAUU) was used to deplete Acinus for the RNA-seq experiment. A second siRNA2 (GCAAGAAGAAGAAGAGCAAUU) was also used for validation. Both siRNAs target all three Acinus isoforms. As a control, cells were transfected with ON-TARGETplus Non-targeting siRNA (Dharmacon). Depletion of eIF4A3 was carried out using siRNA (AGACAUGACUAAAGUGGAA), sequence obtained from (Wang et al. 2014). Cells were collected 48 h after transfection. The following commercial antibodies were used: anti-Acinus antibody (Calbiochem, ab-2 PC552), anti-T7 mouse monoclonal (Novagen, 69522), anti-eIF4A3 antibody (Proteintech, 17504-1-AP), and anti-β-tubulin (Sigma, T4026).

### iCLIP

HeLa cells or HeLa expressing T7-Acinus S* were cultured on a 15-cm dish and irradiated once with 400 mJ/cm^2^ at 254 nm. One-fourth of the 15-cm dish was used for each iCLIP experiment. Three independent iCLIP experiments of T7-Acinus S* were carried out following a published protocol (Konig et al. 2011), with minor modifications. After cell lysis, extracts were sonicated for 5 min (30 sec off and 30 sec on) then centrifuged at 10,000*g* for 3 min. Supernatants were treated with various RNase dilutions. Before the immunoprecipitation, each extract was precleared for 1 h with 10 µL of agarose beads (T7-Acinus S* iCLIP) or Dynabeads protein A (endogenous Acinus iCLIP). For the iCLIP of tagged T7-Acinus S*, a T7 Tag antibody Agarose from Novagen was used for the precipitation in all IPs. For the iCLIP of endogenous protein, the immunoprecipitation (IP) step was carried out using Acinus (Ab-2) PC552 Calbiochem antibody coupled to Dynabeads Protein A, whereas rabbit IgG was used for the control. Dephosphorylation of the RNA 3′ ends was performed on beads using Alkaline Phosphatase, Calf Intestinal (CIP) following the supplier's protocol. The RNA linker (5′Phosphate-UGAGAUCGGAAGAGCGGTTCAG-3′Puromycin) ligated to the 3′ end of the RNAs was described in König et al. (2010). The obtained libraries were purified with Agencourt Ampure XP system (Beckman Coulter) and quantified using High sensitivity DNA ChIP on Agilent 2100 Bioanalyzer. Using different barcodes in the RT primers, the three libraries obtained for T7-Acinus S* iCLIP were sequenced on a single lane while the equivalent control was sequenced on a different lane. Similarly, the four libraries for the endogenous Acinus iCLIP (two replicates for Acinus L and two replicates for Acinus S/S′) were sequenced together with the control libraries on a separate lane. Sequencing was done on an Illumina HiSeq 2000 system at the Beijing Genomics Institute (BGI).

### Read processing

Sequencing data from the iCLIP experiments were processed using mostly the tools on Galaxy server https://usegalaxy.org/. Taking advantage of the iCLIP specific barcodes, identical reads, corresponding to PCR duplicates, were removed. Sequencing artifacts were discarded. Adapter sequences at the 3′ end were removed from the reads using CLIP tool. Barcode splitter tool attributed reads to each replicates. We obtained around 22 million reads for the three T7-Acinus S* experiments, whereas the control experiments produced 4 million reads. All endogenous iCLIP libraries gave 17 million reads (15 million reads for the control). Reads were mapped to the hg19 annotation of the human genome (hg19 assembly) using Bowtie (Langmead et al. 2009) allowing only one mismatch in the seed. Only reads with only one genomic hit were kept. We had a mapping efficiency of 30%–50%, which is explained by the fact that we did not filter reads by length and kept reads mapping to only one genomic location.

### Correlation and annotation

We analyzed the correlation between replicates based on the number of reads obtained for protein-coding genes. Reads mapping to noncoding RNAs and repeats were removed before intersecting the reads with protein-coding genes. Noncoding RNAs, repeats, and protein-coding gene data sets were obtained from the Ensembl GRCh37 annotation and from the hg19 UCSC annotation files (such as RepeatMasker). A correlation plot was produced using the number of reads obtained for each ENSEMBL Id for the different replicates. The correlation was evaluated by calculating the Pearson correlation coefficient.

### Peaks

To identify Acinus binding sites, we used the peak calling algorithm Pyicoclip from the Pyicoteo tools (https://bitbucket.org/regulatorygenomicsupf/pyicoteo) (Althammer et al. 2011). Mapped reads were trimmed to 10 nt and reads overlapping simple and complex repeats as well as known non-coding RNAs were removed for this analysis. As we obtained a good correlation between replicates, we pooled reads from the different replicates. Peaks were called specifically on regions of protein-coding genes. For further analysis, we considered peaks with a modified false discovery rate below 0.01. This analysis was done for each individual iCLIP (after pooling replicates) and the equivalent controls. Peaks detected in the control iCLIP were removed from the Acinus iCLIP peak data. We calculated those significant regions present in two out of the three iCLIP peak sets. Subsequently, reads from all iCLIP experiments that intersected these regions were kept and Pyicoclip was used again with the pooled reads to obtain the final peak set on protein-coding genes.

### Motifs

The peak summit was used as the reference binding site and peaks were ranked based on the number of reads at the summit. We retrieved 20-nt sequences around the summit and searched for motifs on 1000 sequences (top or randomly selected). The search was done for a 6- to 10-nt-long motif using MEME (http://meme-suite.org/tools/meme) (Bailey et al. 2009) allowing up to 20 motifs. For randomly selected peaks, the analysis was done three times with different sets of randomly selected peaks and we confirmed the detection of similar significant motifs in the three searches.

### Comparison with eIF4A3 CLIP

eIF4A3 CLIP-seq data described in Saulière et al. (2012) were downloaded from GEO (GSM1001331) and data were analyzed similarly to the Acinus iCLIP data. After mapping using Bowtie, we used Pyicoclip (Althammer et al. 2011) to identify peaks. We compared the distance between Acinus iCLIP and eiF4A3 CLIP based on the peak summit coordinates.

### Transcriptome mapping

We generated a simplified and a representative transcriptome data set by keeping for each gene the transcript with the longest CDS. First, reads mapping to noncoding RNAs and repeats were discarded. Then, remaining reads were mapped to the sequences of the transcriptome using Bowtie, allowing at most one mismatch and selecting uniquely mapping reads. Peak calling on transcripts was done using Pyicoclip on these mapped reads. Coordinates of the peaks on the transcriptome were converted to genomic coordinates using software available at https://bitbucket.org/regulatorygenomicsupf/pyicoteo. We obtained 29,741 peaks for T7 Acinus S* iCLIP, 23,970 peaks for Acinus L, 32,146 peaks for Acinus S and finally 121,145 peaks for eIF4A3 CLIP. We also defined a list of 13,231 Acinus “common transcriptomic” peaks.

### Peak density in exons and introns

Transcriptomic peaks present in internal exons (first and last exons were removed) were analyzed. Distances between the peak summit and the 5′ and 3′ side of the exon were calculated. The frequency of each distance was evaluated and normalized by the number of analyzed peaks to compare between different data sets. We plotted the density of peaks depending on the distance from the 5′ and 3 exon ends. We also calculated the distance of intronic peak summits from the 5′ and 3′ side of the intron. Finally, histograms showing the number of peak summits depending on the distance were generated.

### Acinus binding to introns using a read count approach

Reads mapping to noncoding RNAs and repeats were discarded. We kept reads that overlap a data set containing all intron interval coordinates (minimum overlap of 5 bp). We calculated for each intron the amount of reads in each iCLIP experiments and the control. We took advantage of the RNA-seq data (described below) to remove introns from very low expressed genes (DESeq normalized count value < 1) and we kept 129,270 introns. We defined CLIP+ introns as introns with fivefold enrichment in Acinus iCLIP versus equivalent control or if there is no read in control, we kept introns with a minimum of five reads. CLIP- introns were defined as introns having a number of reads in all Acinus iCLIPs less or equal to the number of reads in the control iCLIPs.

The strength of the splice site was evaluated on line using the maximum entropy score method (Yeo and Burge 2004) (http://genes.mit.edu/burgelab/maxent/Xmaxentscan_scoreseq.html).

### RNA-seq

RNAs were extracted from Acinus-depleted or control HeLa cells in triplicates. RNA extractions were performed using RNeasy Mini Kit (QIAGEN) with DNase treatment on column following manufacturer's instructions. RNA samples were sent to Beijing Genomics Institute (BGI). The libraries have been obtained using Truseq Transcriptome kit [poly(A) transcript enrichment] and sequenced on Illumina HiSeq providing a minimum of 10 Gb of data per sample (90-bp paired end reads).

### RNA-seq bioinformatics analysis

#### Gene expression

Transcript quantification was done using Sailfish (Patro et al. 2014) on the Ensembl gene annotation (GRCh37). To analyze the expression of a gene, we pooled the estimated read counts obtained by Sailfish for its different transcripts. The differential expression between the control and Acinus-depleted HeLa cell lines was done using DESeq (Anders and Huber 2010). To generate heatmaps, the count values of each gene were first moderated by the variance stabilizing transformation (VST) method using DESeq. We calculated the Euclidean distances among samples and used heatmap2 function of the gplot package to visualize the sample clustering.

#### Splicing

The analysis of splicing changes (for alternative cassette exon and alternative donor and acceptor splice sites) in the RNA-seq of Acinus-depleted cells was done as previously described (Irimia et al. 2014), whereas for analysis of intron retention, we used a previously published intron retention pipeline (Braunschweig et al. 2014).

In differential splicing analysis, stringency criterions for filtering of output include probability (−*r*, the minimal probability of acceptance that is required to consider a comparison to be believable), difference cutoff (−*m*, the minimum difference between two samples that will be accepted such that we are sure with at least probability (0.95 by default) that there is a difference of at least the cutoff (10% by default), and coverage (−*e*, the minimum number of reads for an event to be compared (10 by default). This default is meant to provide a reasonably stringent baseline. The RNA-seq data for eIF4A3, described in Wang et al. (2014), were downloaded from GEO (GSE63091) and data were analyzed similarly to Acinus RNA-seq data. Significant events detected in Acinus or eIF4A3 RNA-seq were compared as described below. We discarded events that cannot be evaluated in both Acinus and eIF4A3. To define a co-regulation, we used a threshold of |Δpsi| > 10 in both data sets. We used BioVenn to create Venn Diagram (Hulsen et al. 2008).

#### Validation of splicing changes

RNA extractions were performed using RNeasy Mini Kit (QIAGEN) following manufacturer's instructions in control cells or cells depleted of Acinus. RNAs were subsequently treated with DNase RQ1 (Promega) to remove any gDNA contamination. cDNA were obtained using Superscript III Reverse Transcriptase (Invitrogen). The PCRs to detect the splicing changes were done with GoTaq Hot Start Colorless Master Mix. The sequences of the used primers can be found in Supplemental Table S4.

#### RNAmap

To investigate whether Acinus directly regulates alternative exons, we looked for Acinus “common” peaks in proximity of the events. We considered a region containing the cassette exon as well as the neighboring constitutive exons. In the case of different possible constitutive exons, we selected the shortest ones. We detected peaks for 31 skipped cassette exons and 30 included cassette exons after Acinus depletion. As a background, we selected 185 alternative cassettes from the list of analyzed exons (percent inclusion between 10 and 90) that are not affected by Acinus (|Δ_psi| < 5) but containing a peak in the event regions. The distribution in exons (50-nt downstream or upstream the splice site) was analyzed plotting a histogram of the normalized counts considering a bin interval of 5 nt. We analyzed the distribution of the relative distance in introns using a normalized count density plot.

#### Intron retention distribution

The intron retention changes upon Acinus depletion was assessed for all, CLIP+ and CLIP− introns. We first removed introns whose intron retention level could not be assessed in some of the RNA-seq samples. For the remaining introns, we calculated the Δpir that corresponds to the change in IR level between Acinus depletion and control and plotted its density distribution. The distribution of the intron retention changes between CLIP+ and CLIP− introns are significantly different as revealed by a Mann-Whitney *U*-test (*P* = 3.4 × 10^−7^, unpaired, greater hypothesis).

#### Gene Ontology enrichment analysis

To find significant GO terms, we used The Database for Annotation, Visualization, and Integrated Discovery (DAVID tool), https://david.ncifcrf.gov/home.jsp. As a background, we provided the list of expressed genes obtained from the RNA-seq (DESeq normalized count value >1).

## DATA DEPOSITION

Raw sequencing data can be found in the Gene Expression Omnibus database with accession number GSE81460. Acinus iCLIP peak files as well as RNA-seq expression data can also be found under this accession.

## SUPPLEMENTAL MATERIAL

Supplemental material is available for this article.

## Supplementary Material

Supplemental Material
