# Supplementary material for: The RNA-binding profile of Acinus, a peripheral component of the exon junction complex, reveals its role in splicing regulation
Source: RNA. 2016 Sep;22(9):1411–26. doi: 10.1261/rna.057158.116 (PMC4986896; doi:10.1261/rna.057158.116)
Supplement: Supplemental Material [file supp_22_9_1411__index.html]

The RNA-binding profile of Acinus, a peripheral component of the exon junction complex, reveals its role in splicing regulation — The RNA-binding profile of Acinus, a peripheral component of the exon junction complex, reveals its role in splicing regulation — Supplemental Material 

# The RNA-binding profile of Acinus, a peripheral component of the exon junction complex, reveals its role in splicing regulation

## Supplemental Material

- Supplemental.pdf
- Supp\_Table\_S1.xlsx
- Supp\_Table\_S4.xlsx
- Supp\_Table\_S2.xlsx
- Supp\_Table\_S3.xlsx
