# Supplementary material for: The RNA-binding profile of Acinus, a peripheral component of the exon junction complex, reveals its role in splicing regulation
Source: RNA. 2016 Sep;22(9):1411–26. doi: 10.1261/rna.057158.116 (PMC4986896; doi:10.1261/rna.057158.116)
Supplement: Supplemental Material [file supp_057158.116_Supplemental.pdf]

Supplementary Material for

The RNA-binding profile of Acinus, a peripheral component of the Exon junction complex, reveals its role in splicing regulation

JULIE RODOR,[1,6] QUN PAN,[2] BENJAMIN J. BLENCOWE,[2,3] EDUARDO EYRAS,[4,5] AND JAVIER F. CÁCERES[1]

[1]MRC Human Genetics Unit, Institute of Genetics and Molecular Medicine, University of Edinburgh, Western General Hospital, Edinburgh EH4 2XU, UK

[2]Donnelly Centre, University of Toronto, Ontario M5S 3E1, Canada

[3]Department of Molecular Genetics, University of Toronto, Toronto, ON M5S 1A8 Canada

[4]Universitat Pompeu Fabra, E08003, Barcelona, Spain

[5]Catalan Institution for Research and Advanced Studies (ICREA), Barcelona, Spain

Table S1. Analysis of read counts in introns for Acinus iCLIP.

Table S2. Significant changes in gene expression in Acinus-depleted cells.

Table S3. Significant alternative splicing changes detected in Acinus-depleted cells.

Table S4. Primer sequences

Supplemental Fig. S1 iCLIP of Acinus protein in HeLa cells.

Supplemental Fig. S2 Annotation of Acinus iCLIP reads.

Supplemental Fig. S3 Peak height for Acinus iCLIP common and unique binding regions.

Supplemental Fig. S4 Annotation and motifs for Acinus L, Acinus S/S' and T7 Acinus S* iCLIP peaks.

Supplemental Fig. S5 Exonic peak distribution for individual Acinus iCLIP experiments.

Supplemental Fig. S6 Exonic distribution of eIFA3 CLIP peaks.

Supplemental Fig. S7 Intronic distribution of individual Acinus and eIF4A3 CLIP peaks.

Supplemental Fig. S8 RNA-seq analysis of Acinus depleted HeLa cells.

Supplemental Fig. S9 The number of reads in introns of LAMB2 and ZMIZ2 correlates with the change in intron retention following Acinus depletion.

Supplemental Fig. S10 A large number of Acinus regulated splicing events are co-regulated by the ECJ core protein, eIF4A3.

Table S1. Analysis of read counts in introns for Acinus iCLIP. This table contains the following sheets: CLIP+ (all iCLIP), CLIP- (all iCLIP) and all introns as well as CLIP+ introns for each individual iCLIP (T7-AciS*, AciL and AciS). The table also contains the change in intron retention level upon Acinus depletion (delta_pir) for each intron in the RNA-seq data and an indication whether this value relies on good quality retention score.

Table S2. Significant changes in gene expression in Acinus-depleted cells. Genes up and down regulated with a minimum 2-fold change and an adjusted *P*-value of 0.05 were kept. The differential expression of the RNAseq data was analyzed using DESeq. The table gives the normalized read counts in si-Acinus samples versus si-Control, the fold change and the calculated *P*-value.

Table S3. Significant alternative splicing changes detected in Acinus-depleted cells. The table gives the gene name, ENSEMBL Id, an event Id, the full coordinates of the events, the percent splice in each individual replicate as well as the change between si-Acinus and si-Control (delta_psi). We also indicated whether this event is regulated by eIF4A3 (co-reg: co-regulation, opp: change in splicing but in opposite direction, no: no co-regulation, NA: no reliable value in eIF43 RNA-seq).

Table S4. Primer sequences

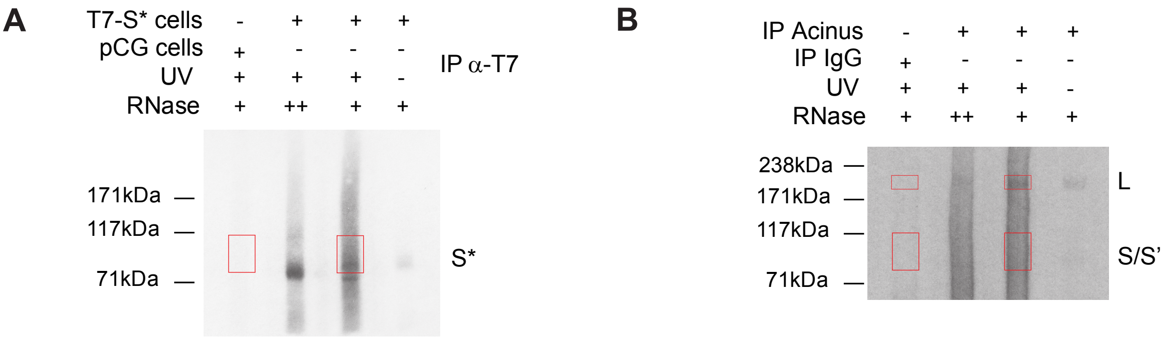

C

number of processed reads

	iCLIP T7-Acinus*	iCLIP control pCG
rep1	12,473,634	366,303
rep2	7,054,149	2,474,832
rep3	2,377,232	1,091,978

	iCLIP Acinus L	iCLIP control IgG
rep1	6,343,824	5,090,499
rep2	4,144,048	1,207,348

	iCLIP Acinus S	iCLIP control IgG
rep1	5,423,270	6,065,982
rep2	1,568,815	2,656,850

D

number of mapped reads

	iCLIP T7-Acinus*	iCLIP control pCG
rep1	5,162,706	151,869
rep2	2,244,578	70,212
rep3	766,715	187,916

	iCLIP Acinus L	iCLIP control IgG
rep1	2,305,543	1,289,710
rep2	2,090,547	88,215

	iCLIP Acinus S	iCLIP control IgG
rep1	2,693,943	1,954,877
rep2	623,991	435,962

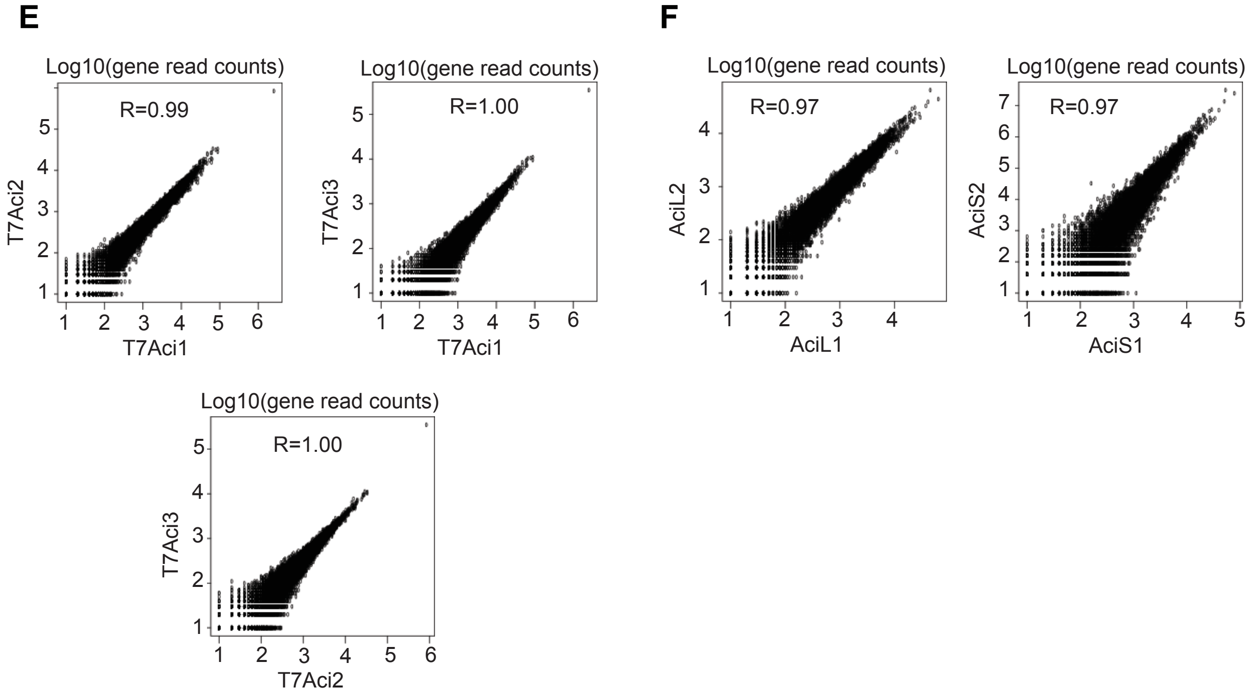

Supplemental Fig. S1. CLIP of Acinus protein in HeLa cells. (A-B) iCLIP experiment for T7-Acinus S* iCLIP (A) and endogenous Acinus iCLIP (B). Autoradiograph of crosslinked protein/RNA complexes after immunoprecipitation and 32P RNA labeling. RNA was partially digested with Low (+) or High(++) RNase I concentration. Sections of the membrane cut for the library preparation are indicated by red boxes. Control experiment for T7-Acinus S* iCLIP corresponds to cells transfected with an empty vector (pCG) followed by an immunoprecipitation using T7 Tag antibody Agarose. Control experiment for endogenous Acinus iCLIP corresponds to an immunoprecipitation using rabbit IgG. (C) Number of reads obtained for each replicates of the three iCLIP experiments after processing of the sequencing data (removal of PCR duplicates, trimming of adapter sequences, attribution of the read to one specific replicate using the barcode). (D) Number of reads mapped to human genome (hg19 assembly) using Bowtie (only 1 mismatch permitted in the seed and 1 hit allowed) for each replicates of the three iCLIP experiments. (E) Analysis of the correlation between replicates T7_Aci1, T7_Aci2 and T7_Aci3 of the tagged T7 Acinus S* iCLIP experiment for the number of read counts (Log10) per gene. The Pearson correlation coefficient for each comparison is shown. (F) Analysis of the correlation between replicates 1 and 2 of the endogenous Acinus L and S iCLIP experiment considering the number of read counts (Log10) per gene. The Pearson correlation coefficient for each comparison is shown.

A

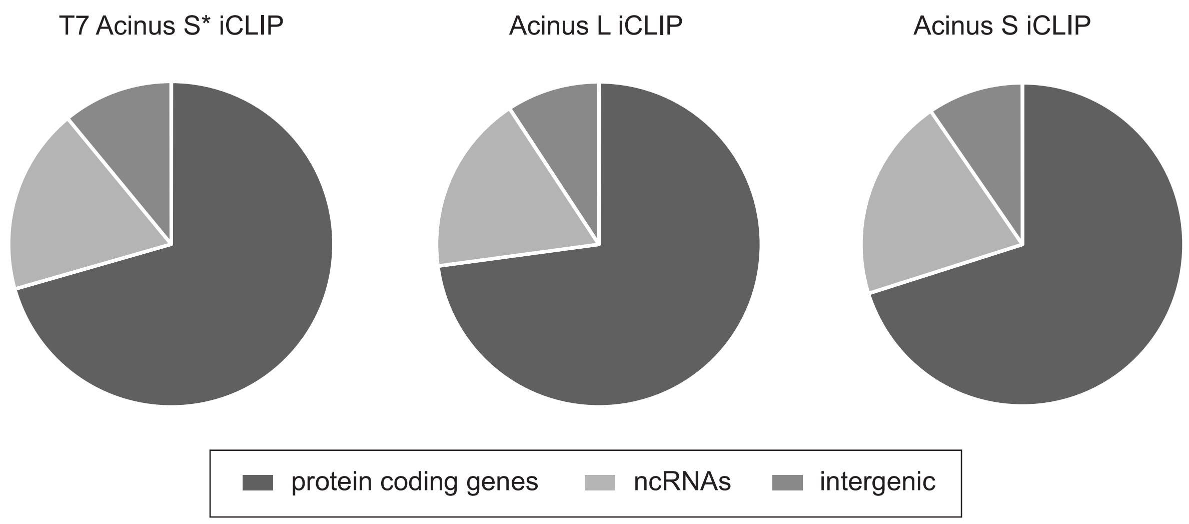

B

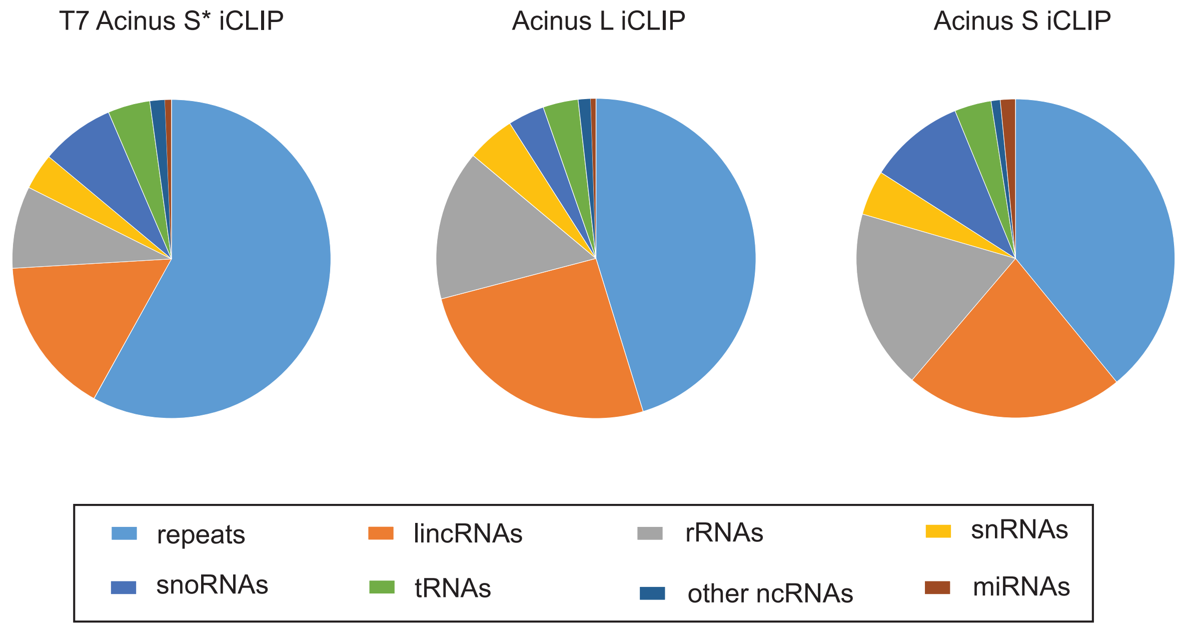

Supplemental Fig. S2. Annotation of Acinus iCLIP reads. (A) Distribution of T7 Acinus S*, Acinus L and Acinus S iCLIP reads among protein-coding genes, non-coding RNAs and intergenic regions. (B) Proportion of reads mapping to various non-coding RNAs for T7 Acinus S*, Acinus L and Acinus S iCLIP. Non-coding RNAs, repeats and protein coding gene datasets were obtained from the Ensembl GRCh37 annotation and from the hg19 UCSC annotation files.

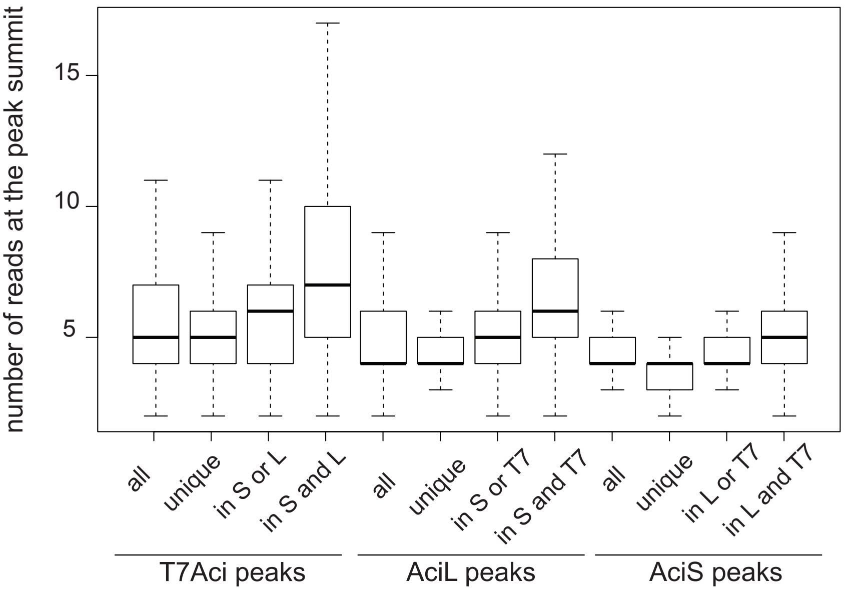

Supplemental Fig. S3. Peak height for Acinus iCLIP common and unique binding regions Box plot showing the number of reads at the peak summit for iCLIP peaks unique or common to other iCLIP experiments. ‘T7’ refers to T7 Acinus S* iCLIP while ‘L‘ and ‘S’ correspond to endogenous Acinus L and S iCLIP.

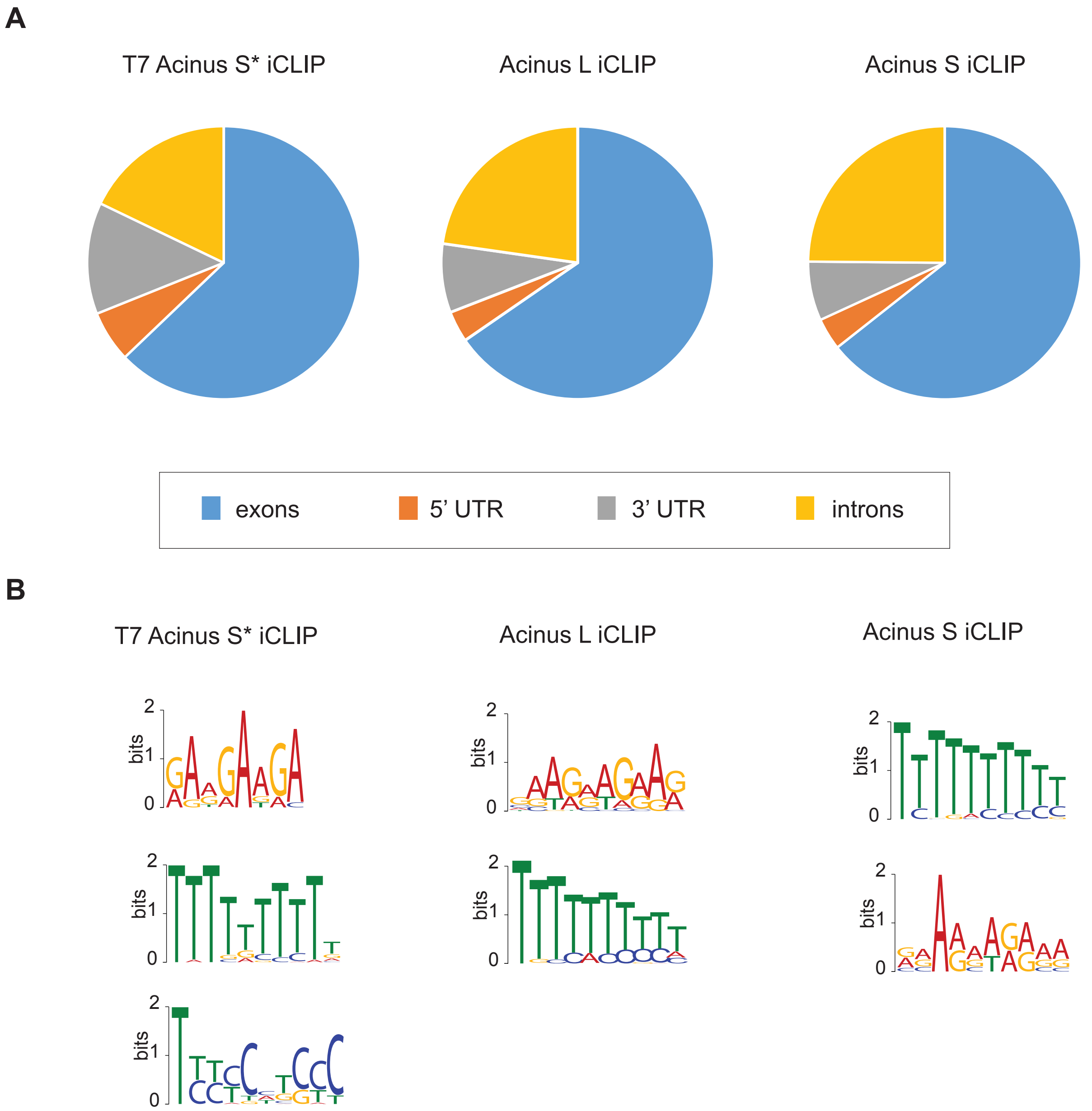

Supplemental Fig. S4. Annotation and motifs for Acinus L, Acinus S/S' and T7 Acinus S* iCLIP peaks. (A) Distribution of Acinus binding peaks among coding exons, 5'UTR, 3'UTR and introns. (B) Significant motifs found using MEME tool (search for 6-10nt motif) in 20nt sequences centered on the peak summit from the top 1000 peaks.

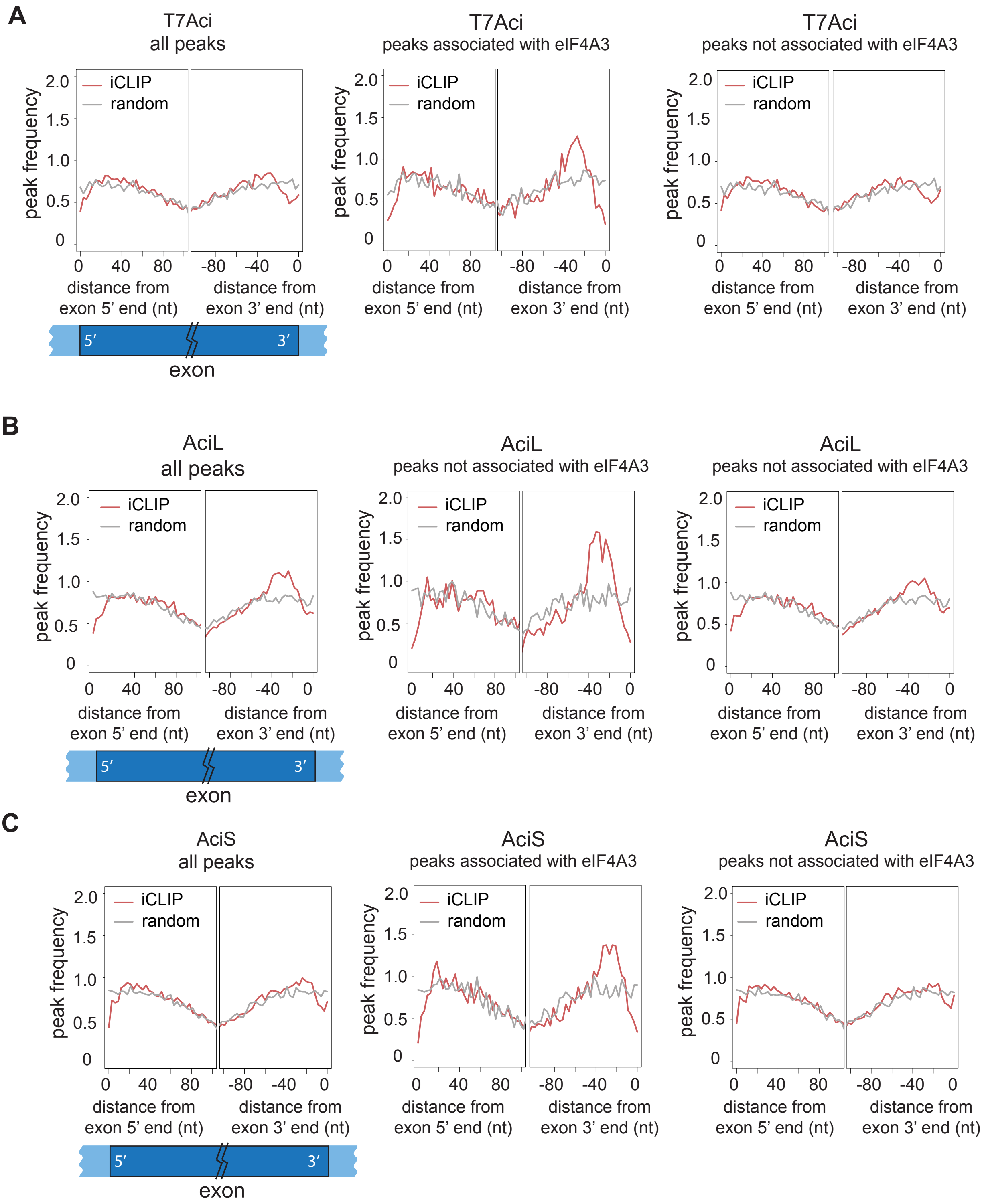

Supplemental Fig. S5. Exonic peak distribution for individual Acinus iCLIP experiments. (A-C) Distribution of the peak frequency according to the distance of the peak summit from the exon 5' end or the exon 3' end. Only internal exons were used. The iCLIP data is represented by the red line. The grey line corresponds to randomized peak summit positions. (A) Analysis of Acinus L 'transcriptomic' peak data for all peaks (n=19,166), peaks associated with eIF4A3 peaks (n=3,281) or peaks not associated with eIF4A3 (n=15,885). (B) Analysis of Acinus S 'transcriptomic' peak data for all peaks (n=25,568), peaks associated with eIF4A3 peaks (n=4,137) or peaks not associated with eIF4A3 (n=21,431). (C) Analysis of T7 Acinus S* 'transcriptomic' peak data for all peaks (n=20136), peaks associated with eIF4A3 peaks (n=3537) or peaks not associated with eIF4A3 (n=16599). eIF4A3 CLIP-seq data described in Sauliere et al, 2012 were downloaded from [GEO:GSM1001331].

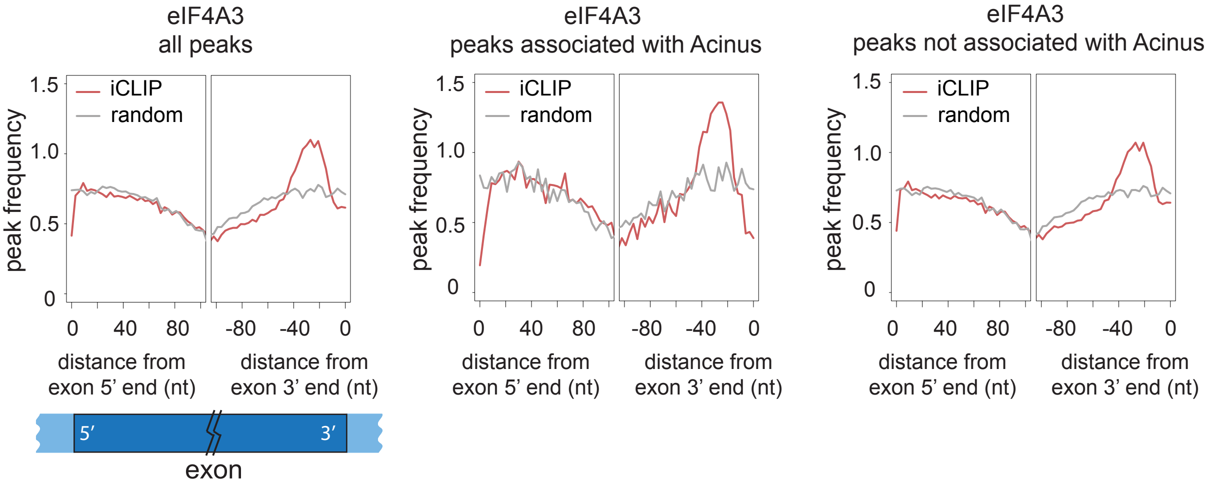

Supplemental Fig. S6. Exonic distribution of eIFA3 CLIP peaks. Distribution of the peak frequency according to the distance of the peak summit from the exon 5' end or the exon 3' end. This analysis was done using eIF4A3 'transcriptomic' peak data obtained using eIF4A3 CLIP-seq data described in (Saulière et al., 2012)and downloaded from [GEO:GSM1001331]. We analyzed all peaks (n=88,150), peaks associated with Acinus peaks (any of the Acinus iCLIP experiments) (n=9,090) or peaks not associated with Acinus (n=79,060). Only internal exons were used. The CLIP data is represented by a red line. The grey line corresponds to randomized peak summit positions.

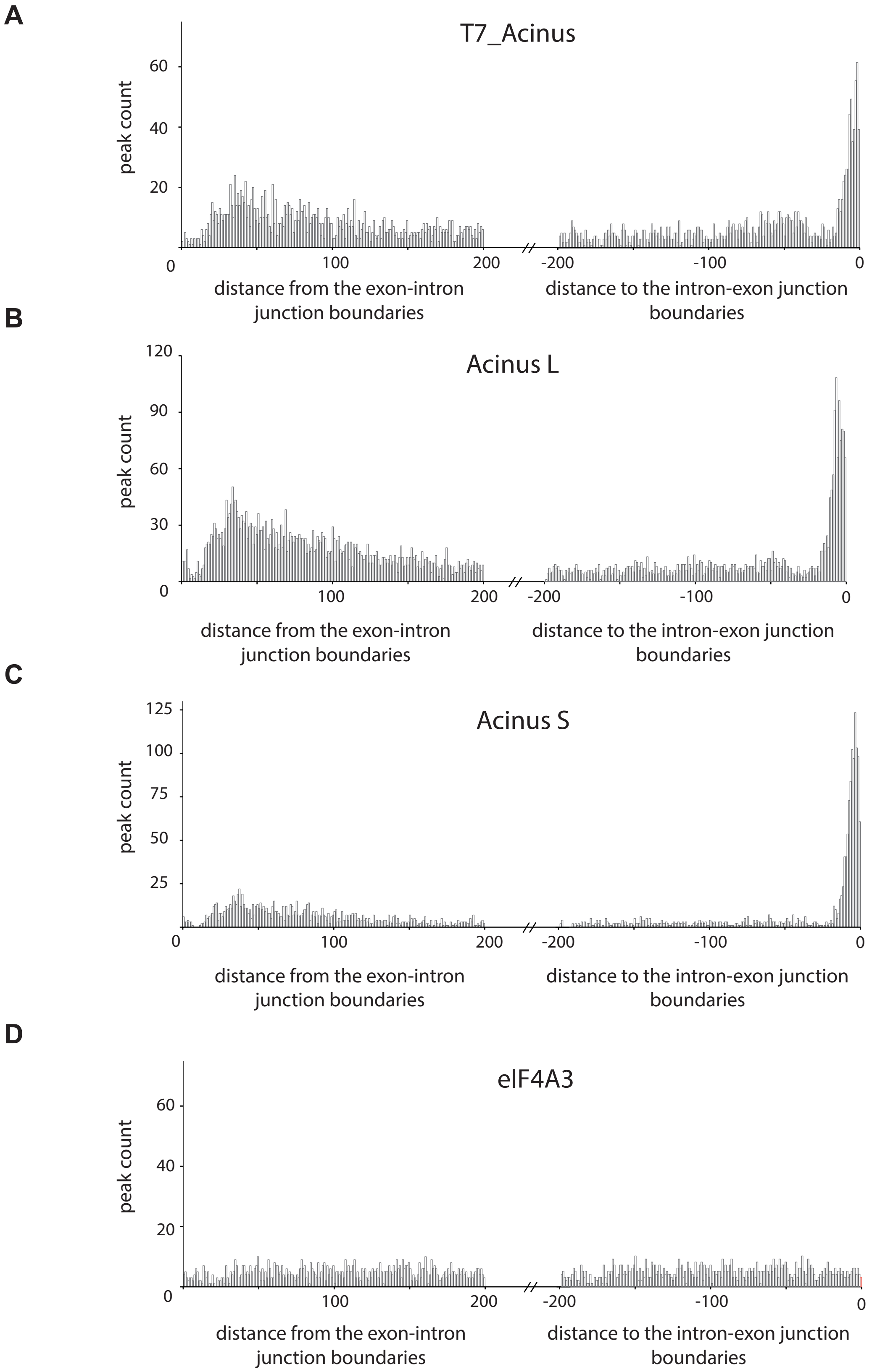

Supplemental Fig. S7. Intronic distribution of individual Acinus and eIF4A3 CLIP peaks. Histogram showing the number of peaks in introns depending on the distance of the peak summit from and to the exon-intron junction boundaries for T7-Acinus S* (A), Acinus L (B), Acinus S (C) iCLIP and eIF4A3 CLIP (D).

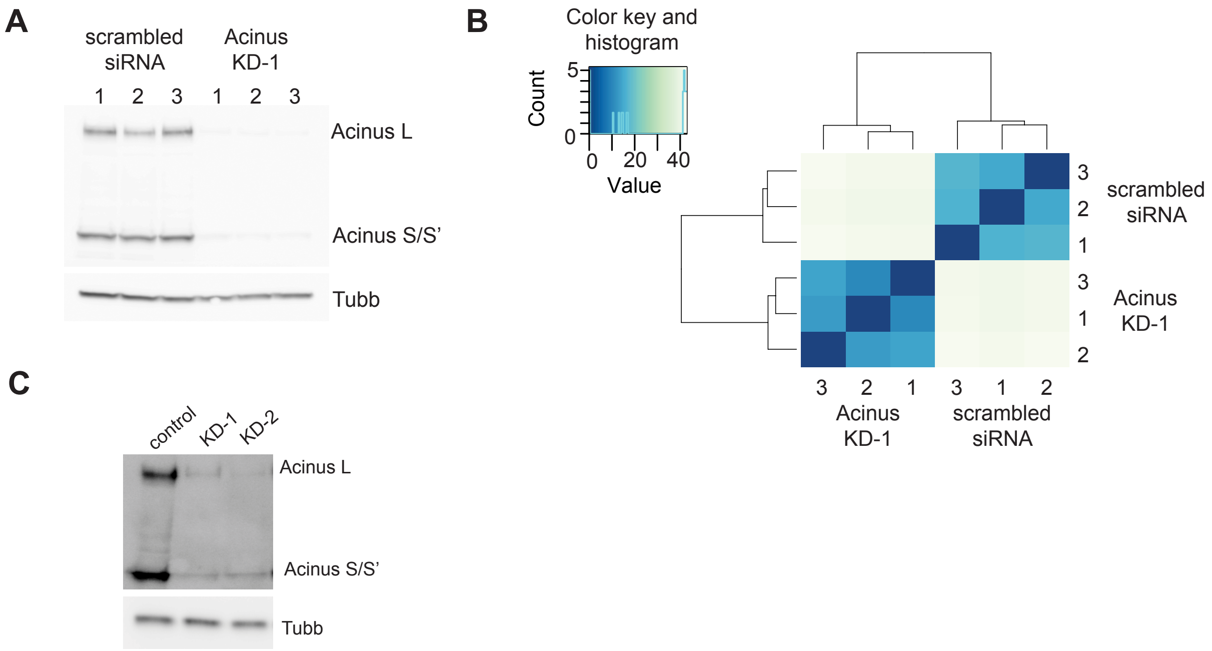

Supplemental Fig. S8. RNA-seq analysis of Acinus depleted HeLa cells. HeLa cells were treated with a control non-targeting siRNAs or an siRNA specific for the three Acinus isoforms (triplicates). (A) Western blot of Acinus depletion in HeLa cells in samples used for the RNA-seq analysis. (B) Heatmap showing the Euclidean distances between the RNA-seq samples as calculated from the variance stabilising transformation of the count data using DESeq. The count data was obtained after mapping and transcript quantification using Sailfish (Patro et al, 2014). (C) Depletion of Acinus in HeLa cells, using two different siRNAs, was analyzed by Western blotting with a specific antibody.

A

LAMB2 gene: chr3 strand -

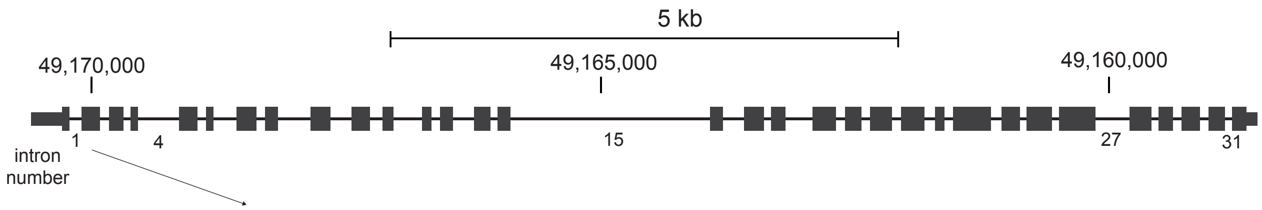

intron number	1	2	3	4	5	6	7	8	9	10	11	12	13	14	15	16	17	18	19	20	21	22	23	24	25	26	27	28	29	30	31
read count in Acinus iCLIP	7	4	0	18	0	3	2	16	12	9	108	2	4	3	8	0	19	63	8	0	7	1	6	6	32	34	62	0	0	0	8
delta_PIR between Acinus KD and control	4	1	0	43	2	0	0	7	4	10	38	4	2	3	1	0	11	48	9	0	3	1	0	1	15	13	34	0	0	1	-3

B

ZMIZ2 gene: chr7 strand +

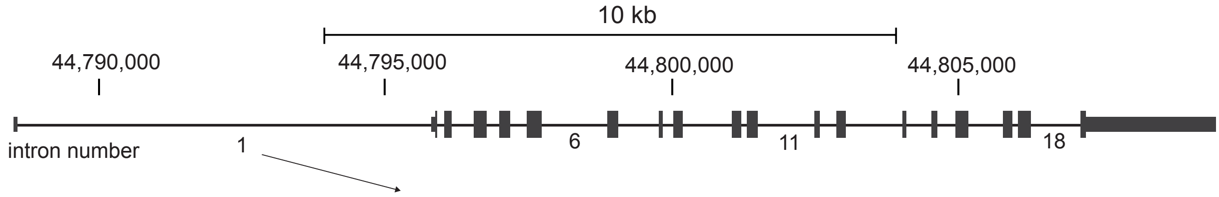

intron number	1	2	3	4	5	6	7	8	9	10	11	12	13	14	15	16	17	18
read count in Acinus iCLIP	73	3	34	4	22	1	311	127	18	19	55	31	11	5	7	237	1	29
delta_PIR between Acinus KD and control	0	0	9	-1	0	0	36	22	2	1	0	0	0	0	0	46	0	0

C

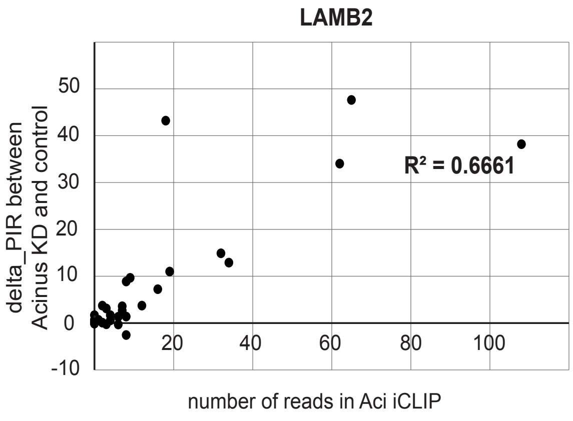

D

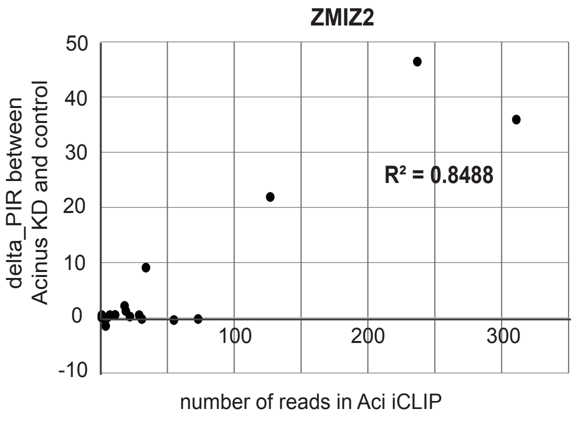

Supplemental Fig. S9. The number of reads in introns of LAMB2 and ZMIZ2 correlates with the change in intron retention following Acinus depletion. (A-B) Structure of LAMB2 (A) and ZMIZ2 (B) gene. The table below the structure indicates for each intron, the number of reads in Acinus CLIP (after removing reads obtained in the control) with the change of intron level obtained in the RNA-seq analysis (delta_PIR: change of Percent Intron Retention between Acinus depletion and control). Introns characterized by a large number of reads and showing a change in their intron retention level are highlighted in orange. (C-D) Plot showing the correlation between the change of intron level and the iCLIP number of reads for LAMB2 (C) and ZMIZ2 gene (D). The Pearson correlation coefficient is indicated.

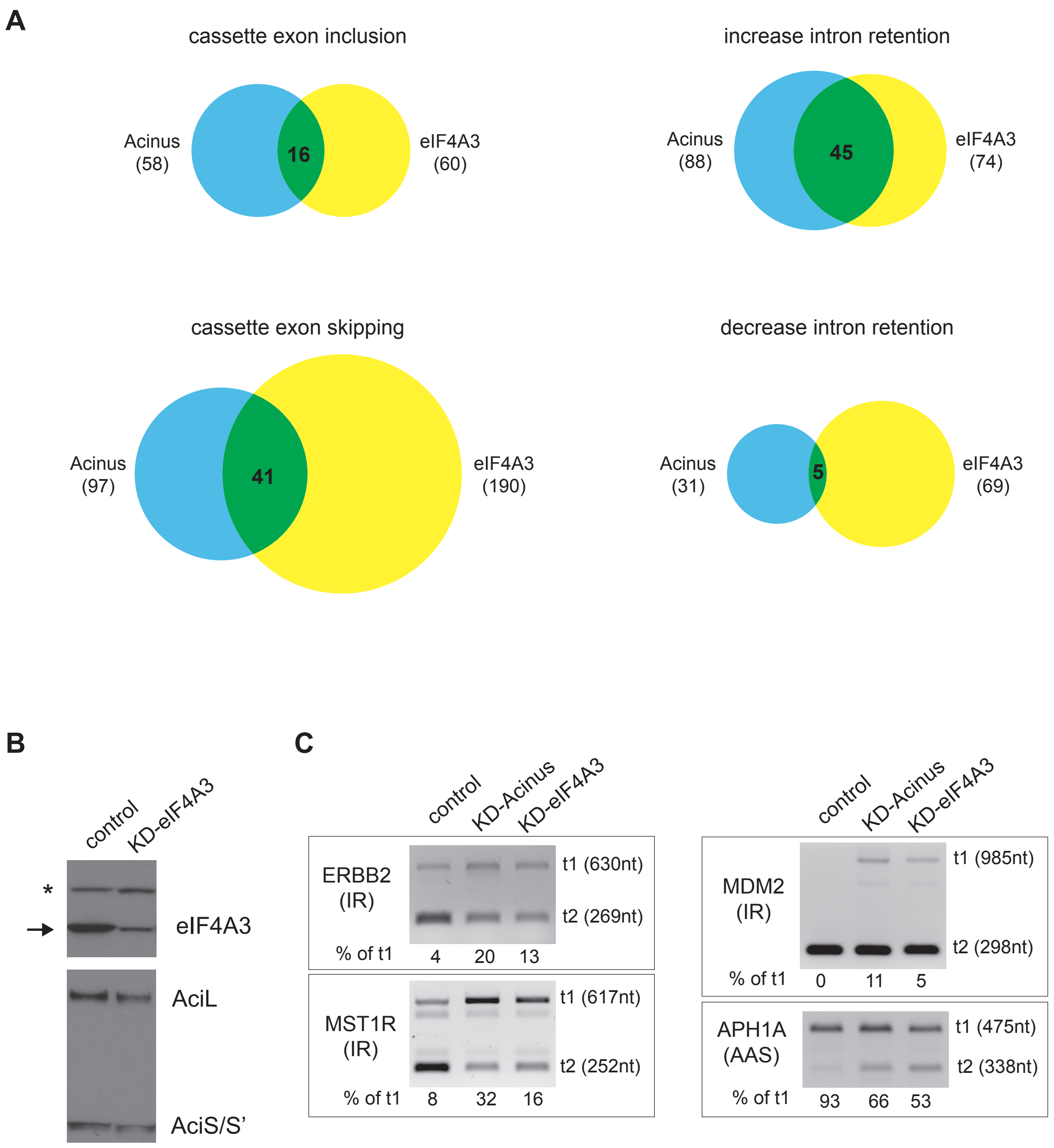

Supplemental Fig. S10. A large number of Acinus regulated splicing events are co-regulated by eIF4A3. (A) Venn Diagram showing the overlap between Acinus and eIF4A3 regulated splicing events. The eIF4A3 RNA-seq dataset previously described (Wang et al, 2014) were downloaded from GEO (GSE63091). The analysis was done using the same pipeline as used for Acinus RNA-seq. Events regulated by eIF4A3 or Acinus were compared. We discarded events with no value in the other dataset and defined co-regulated events as events showing a change in the same direction with a delta_psi above 10 (see methods). (B) Western blot of eIF4A3 depleted HeLa cells. Cells were collected after 48h of transfection using a specific siRNA. Detection of Acinus and eIF4A3 proteins using specific antibodies. The arrow in eIF4A3 western indicates the specific eIF4A3 band while the non-specific band is indicated by an asterisk. (C) Validation of co-regulated splicing events in Acinus and eIF4A3-depleted HeLa cells by RT-PCR. We detected the intron retention of ERBB2, MST1R and MDM2 genes as well as the alternative acceptor splice site of APH1A gene.
